# Immunoaffinity‐Mimetic Assembly of Peptide‐Aptamer Conjugates and Stem Cell‐Derived Exosomes into Hierarchical Microgels for Spinal Cord Injury Repair

**DOI:** 10.1002/advs.202519701

**Published:** 2026-01-09

**Authors:** Dantong Zheng, Yuxin Bai, Zhibo Liu, Zinan Zhao, Yiming Kong, Yao Xu, Qi Jia, Rongrong Zhu, Yong Hu

**Affiliations:** ^1^ Department of Polymeric Materials School of Materials Science and Engineering Tongji University Shanghai China; ^2^ Department of Orthopedics, School of Life Sciences and Technology Key Laboratory of Spine and Spinal Cord Injury Repair and Regeneration of Ministry of Education Tongji Hospital Affiliated to Tongji University Tongji University Shanghai China; ^3^ Department of Orthopedic Oncology Shanghai Changzheng Hospital Second Affiliated Hospital of Naval Medical University Shanghai China

**Keywords:** mesenchymal stem cell‐derived exosomes, microgel, neuroregeneration, peptide‐aptamer conjugates, spinal cord injury

## Abstract

Mesenchymal stem cell‐derived exosomes (MSC‐Exo) containing various paracrine mediators have recently been recognized to promote functional recovery of spinal cord injury (SCI). However, their biomedical applications are hindered by limited possibilities for specific integration within the delivery systems. In this study, inspired by antibody‐antigen binding, we present an unprecedented assembly strategy that leverages immunoaffinity‐mimetic interactions of peptide‐aptamer (Peptide‐Apt_CD63_) conjugates targeting CD63 markers on MSC‐Exo, yielding a hierarchical microstructure to enhance SCI repair. The conjugate, serving as the microgel scaffold, is synthesized by grafting azide‐modified Apt_CD63_ onto poly(propargyl cysteine‐*co*‐γ‐propargyl‐_L_‐glutamate). It exhibits excellent antioxidant capability to mitigate oxidative stress and immune responses at the injury site. In addition, this conjugate constrains the tethered MSC‐Exo within the lesion site during administration, yet its nuclease susceptibility allows for the release of MSC‐Exo to promote the proliferation and migration of neural stem cells and to direct their differentiation into neurons rather than astrocytes. These synergistic effects ultimately enhance neuroregeneration, thereby promoting the motor function recovery of SCI mice. Our work represents a pioneering effort in harnessing the specificity of immunoaffinity‐mimetic interactions between aptamers and biomacromolecules for the high‐performance assembly of chemically vulnerable biologics, facilitating widespread applications across materials science and life science.

## Graphical abstract

Inspired by antibody‐antigen binding, this study develops an unprecedented immunoaffinity‐mimetic assembly strategy, with Peptide‐Apt_CD63_ conjugates acting as antibody surrogates binding CD63 epitopes on mesenchymal stem cell‐derived exosomes. This creates a hierarchical microstructure intended to synergistically integrate antioxidative and anti‐inflammatory strategies with neuroregenerative processes to enhance spinal cord injury repair.

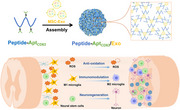

## 1 Introduction

As a devastating disorder of the central nervous system, spinal cord injury (SCI) caused by acute mechanical trauma often results in permanent locomotor and sensory dysfunctions, imposing burdens on both patients and society [1, 2]. The lesion site is characterized by neuronal apoptosis, glial scar formation, and demyelination, in addition to severe oxidative stress and immune response [3, 4]. Among various therapeutic modalities, the transplantation of mesenchymal stem cell‐derived exosomes (MSC‐Exo) containing various paracrine factors demonstrates superior efficacy in lesion regeneration and functional recovery following SCI [5, 6]. Even though MSC‐Exo have garnered increasing attention for their potential in SCI repair by promoting neuronal differentiation and myelin regeneration, as well as reducing glial scar formation [7, 8, 9], in vivo short retention and fast clearance of such small nanoscale vesicles impairs the therapeutic efficacy. To resolve these issues, various conventional polymers, such as gelatin [5, 10], hyaluronic acid [6, 11], poly(ethylene glycol) [12, 13], etc., have been reported to encapsulate Exo within their 3D crosslinked networks. However, the incorporation of Exo during the chemical reaction‐driven crosslinking processes may compromise their bioactivity, and loading Exo post‐gelation often fails to achieve high loading efficiency and stable retention. Therefore, it is crucial to establish a mild yet effective approach for integrating Exo with the polymeric scaffold of delivery systems.

Peptide‐oligonucleotide conjugates represent a class of momentous macromolecules that consist of a polypeptide segment covalently linked to a nucleic acid component [14, 15]. These conjugates integrate the properties of both peptides and oligonucleotides, allowing for arbitrary predefined sequences and structural configurations. Therefore, they emerge as distinctive building blocks for the de novo design of scale‐spanning architectures that cannot be realized using peptides or oligonucleotides alone [16, 17, 18]. Through systematic evolution of ligands by exponential enrichment (SELEX), functional oligonucleotides known as aptamers can be effectively programmed to bind target molecules with exceptional specificity and affinity [19, 20]. This binding mechanism parallels the interactions observed in antibody‐antigen complexes, yet aptamers offer several advantages, including simpler generation, enhanced chemical stability, reduced immunogenicity, lower production cost, and minimized batch‐to‐batch variability compared to antibodies [21]. In this context, nucleic acid aptamers have been exploited to selectively enrich Exo by specifically binding to the membrane proteins on their surface under physiological conditions [22, 23, 24]. Further considering that peptide‐aptamer conjugates incorporate multiple aptamer ligands and preserve the exceptional specificity and affinity of aptamers for protein receptors [25, 26], there is great anticipation for proposing an immunoaffinity‐mimetic strategy to specifically and steadily assemble peptide‐aptamer conjugates with MSC‐Exo under mild conditions and with high utilization efficiency by mimicking antibody‐antigen binding.

**SCHEME 1:**
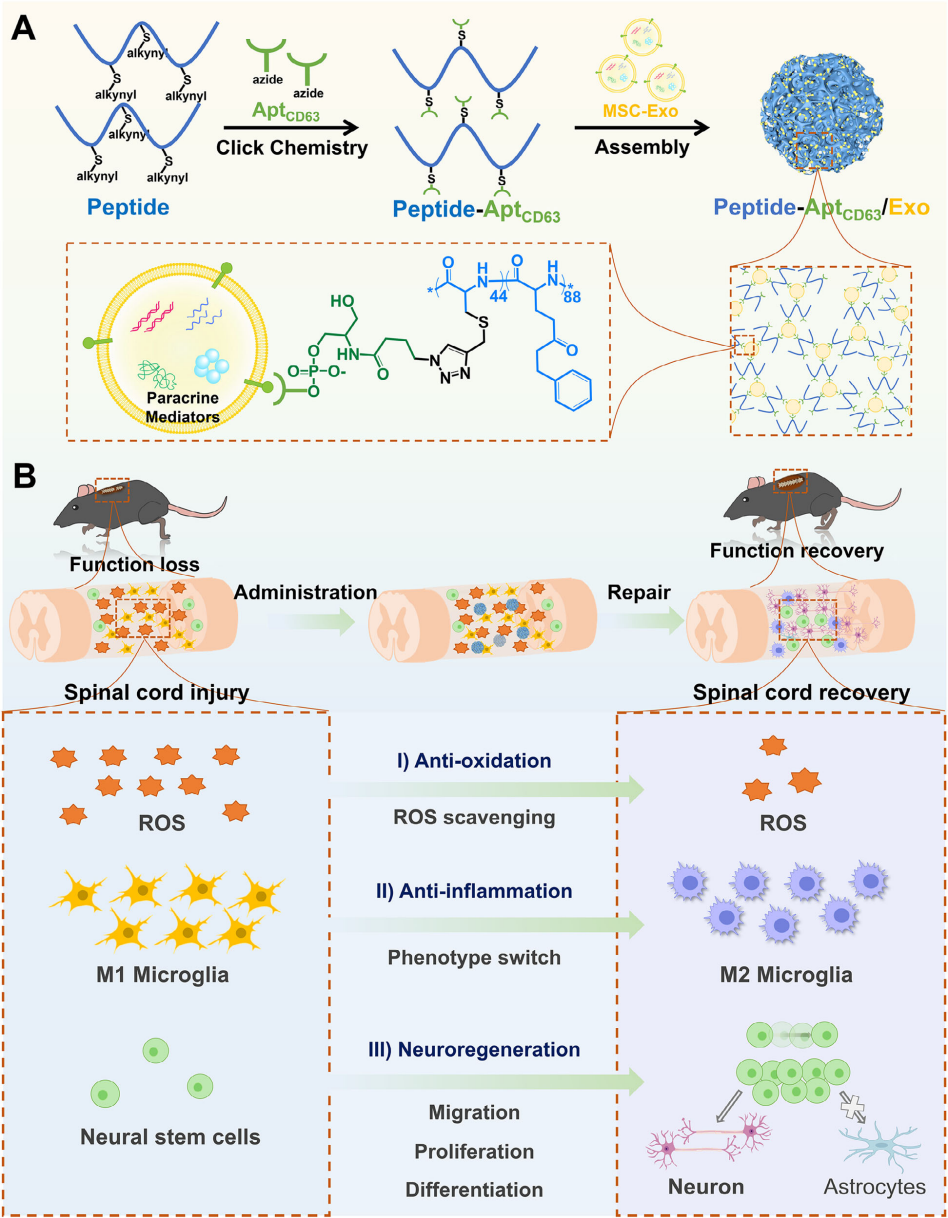
Synthesis of Peptide‐Apt_CD63_/Exo microgels for SCI repair. (A) Schematic illustration depicting the synthesis process of Peptide‐Apt_CD63_/Exo microgel. (B) Overview of the therapeutic mechanisms employed by the Peptide‐Apt_CD63_/Exo microgels in the SCI repair.

In this study, a copolymer polypeptide, poly(propargyl cysteine‐*co*‐γ‐benzyl‐_L_‐glutamate), is synthesized via the ring‐opening polymerization (ROP) of amino acid N‐carboxyanhydride (NCA) monomers, followed by the grafting of an azido group‐modified CD63 aptamer (Apt_CD63_) to yield the Peptide‐Apt_CD63_ conjugate. Given the abundant presence of CD63 proteins on each MSC‐Exo, these particles act as crosslinkers, interacting with Apt_CD63_ in a manner akin to an antibody‐antigen binding mechanism. This interaction results in the formation of a 3D crosslinked architecture, designated as the Peptide‐Apt_CD63_/Exo microgel. Upon implantation of the microgel into the injury site of a SCI mouse model, the presence of thioether groups within the polypeptide facilitates the effective clearance of reactive oxygen species (ROS), thereby alleviating the oxidative stress and the inflammatory responses. The synergistic anti‐inflammatory effect of Peptide‐Apt_CD63_ conjugate in conjunction with MSC‐Exo significantly promotes the transition of M1‐type microglia to the M2 phenotype. Furthermore, the nuclease susceptibility of the conjugate allows for the release of MSC‐Exo to promote the proliferation and migration of neural stem cells (NSCs), as well as the differentiation of NSCs into mature neurons rather than astrocytes. These effects ultimately lead to effective neuroregeneration at the lesion site, thereby promoting the recovery of motor function of SCI mice (Scheme 1). Importantly, this study represents significant conceptual advancements in immunoaffinity‐mimetic assembly of peptide‐oligonucleotide conjugates and vulnerable biologics into a hierarchical architecture, which adds an additional dimension for widespread applications across materials science and life science.

## 2 Results and Discussion

### 2.1 Assembly and Characterization of Peptide‐Apt_CD63_/Exo Microgels

**FIGURE 1:**
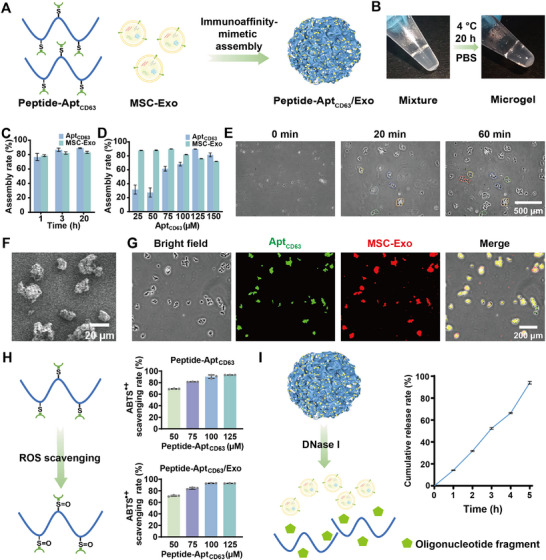
Characterization of Peptide‐Apt_CD63_/Exo microgels. (A) Schematic illustration of the Peptide‐Apt_CD63_/Exo assembly. (B) Photographs of the mixture of Peptide‐Apt_CD63_ (125 µm Apt_CD63_) and MSC‐Exo (5.0 × 10^5^ particles/µL) before and after assembly. (C) Assembly rates of peptide‐tethered Apt_CD63_ (125 µm Apt_CD63_) and MSC‐Exo (5.0 × 10^5^ particles/µL) at different time points. (D) Assembly rates of peptide‐tethered Apt_CD63_ and MSC‐Exo with different concentrations of Apt_CD63_ (25–150 µm) and a constant concentration of MSC‐Exo (5.0 × 10^5^ particles/µL) after 20 h co‐incubation. (E) Time‐lapse images of the mixture of Peptide‐Apt_CD63_ and MSC‐Exo from 0 min to 60 min. (F) SEM image of Peptide‐Apt_CD63_/Exo microgels. (G) Fluorescence microscopy images of Peptide‐Apt_CD63_/Exo microgels. (H) Schematic illustration of the ROS scavenging mechanism of Peptide‐Apt_CD63_, and ABTS^•+^ scavenging rates of Peptide‐Apt_CD63_ and Peptide‐Apt_CD63_/Exo microgel at different concentrations of Peptide‐Apt_CD63_. (I) Schematic illustration of nuclease‐triggered MSC‐Exo release from microgels, and cumulative release profile of MSC‐Exo after incubation of microgels with DNase I (5 U mL^−1^) up to 5 h. All data are represented by mean ± SD (n = 3, ^*^ *p* < 0.05, ^**^ *p* < 0.01, ^***^ *p* < 0.001, ^****^ *p* < 0.0001).

First, a copolymer polypeptide with alkynyl groups was synthesized via ROP of propargyl cysteine NCA (PRC‐NCA) and γ‐benzyl‐_L_‐glutamate NCA (BLG‐NCA) monomers (Figure S1). Each polypeptide chain was determined to contain approximately 44 PRC and 88 BLG units, respectively, according to ^1^H NMR (Figure S2) and GPC analyses (Figure S3). Subsequently, the peptide‐oligonucleotide conjugate, also named Peptide‐Apt_CD63_, was synthesized through grafting azido group‐modified Apt_CD63_ onto a polypeptide with alkynyl groups via click chemistry (Figures S1 and S4). The average number of Apt_CD63_ moieties grafted onto each polypeptide chain approached ∼3.0, as determined by measuring the unbonded Apt_CD63_ with a microspectrophotometer after ultrafiltration. Ultimately, by simply mixing the synthesized Peptide‐Apt_CD63_ with Exo in a physiological buffer solution, the specific molecular interaction between Apt_CD63_ ligands on the conjugate and the CD63 receptors expressed on MSC‐Exo induced the formation of the Peptide‐Apt_CD63_/Exo microgel (Figure 1A).

This immunoaffinity‐mimetic assembly of microgels led to a clear phase separation from the uniform reaction mixture (Figure 1B), with high assembly rates for both peptide‐tethered Apt_CD63_ (∼77%) and MSC‐Exo (∼79%) within 1 h, at the Apt_CD63_ concentration of 125 µm and MSC‐Exo concentration of 5.0 × 10^5^ particles/µL (Figure 1C). Subsequently, we investigated the utilization efficiency of peptide‐tethered Apt_CD63_ and MSC‐Exo with different concentrations of Apt_CD63_ (25–150 µm) at a constant concentration of MSC‐Exo (5.0 × 10^5^ particles/µL). We found that the assembly rate of MSC‐Exo consistently maintained a high level (>72%) after 20 h of incubation with Peptide‐Apt_CD63_ with various concentrations, while that of Peptide‐Apt_CD63_ increased with rising concentration, reaching up to ∼90% at 125 µm Apt_CD63_ (Figure 1D). Similar assembly rates and phase separation characteristics were observed in the assembly of Peptide‐Apt_CD63_ with negatively charged Milk‐Exo expressing the CD63 protein (Figures S5–S7). Nevertheless, Milk‐Exo at an equivalent concentration of 5.0 × 10^5^ particles/µL failed to assemble into microgels under an identical condition to MSC‐Exo (Figure S8). This failure is attributable to the substantially lower CD63 expression in Milk‐Exo compared to MSC‐Exo, as demonstrated by ELISA quantification (Figure S9). This finding suggests that the immunoaffinity‐mimetic assembly strategy can be generalized to type of Exo that express specific target molecules, such as CD63, as demonstrated in this study. The assembly process of Peptide‐Apt_CD63_ and Exo into Peptide‐Apt_CD63_/Exo microgels with the increase of time was monitored by time‐lapse imaging, which clearly showed the appearance and size increase of microassemblies (Figure 1E).

Unless stated otherwise, Peptide‐Apt_CD63_/Exo microgels were synthesized through co‐incubation of Peptide‐Apt_CD63_ (125 µm Apt_CD63_) and Exo (5.0 × 10^5^ particles/µL) at 4 °C for 20 h for further studies. The morphology of the thus‐formed microgels was observed using scanning electron microscopy (SEM) at larger magnification, revealing a distinct 3D hierarchical network (Figure 1F; Figure S10). This structure contrasts with the morphologies observed for the Peptide‐Apt_CD63_ membrane and MSC‐Exo aggregates (Figure S11). The mean particle size of the microgels was ∼30.34 µm (Figure S12). The co‐existence of Peptide‐Apt_CD63_ and MSC‐Exo within the microgels was confirmed by fluorescence microscopy, as evidenced by the clear observation of green fluorescence from SYBR Green II staining of Peptide‐Apt_CD63_ and red fluorescence from CM‐Dil staining of MSC‐Exo (Figure 1G). Furthermore, Peptide‐Apt_CD63_ conjugate containing the antioxidant thioether [27, 28, 29] exhibited exceptional antioxidant capacity, effectively scavenging the ABTS^•+^ radical, thereby imparting significant antioxidant properties to the microgels (Figure 1H). Due to the nuclease susceptibility of the nucleic acid, MSC‐Exo could be readily released from Peptide‐Apt_CD63_/Exo microgels upon exposure to DNase I (Figure 1I). Interestingly, this nuclease‐triggered release from microgels preserved the structural integrity and CD63 level of Exo, comparable to pre‐assembly Exo (Figures S13 and S14). Furthermore, we found that cumulative MSC‐Exo release remained below 8% in normal saline, whereas in FBS it surpassed 60% within 3 h due to the presence of nuclease (Figure S15).

### 2.2 Cellular Internalization, In Vitro Antioxidation, Immunomodulation, NSC Proliferation, and NSC Migration

**FIGURE 2:**
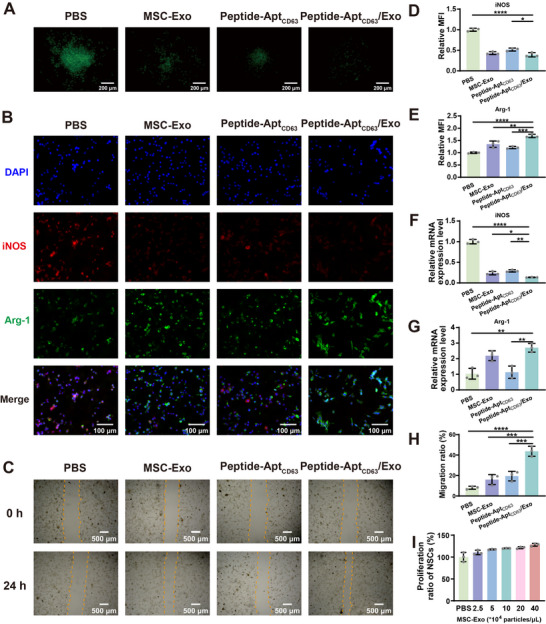
In vitro antioxidation, immunomodulation, NSC proliferation, and NSC migration. (A) Fluorescence microscopy images of intracellular ROS using an ROS indicator, DCFH‐DA, in NSCs after different treatments (Peptide‐Apt_CD63_, 37.4 µm; MSC‐Exo, 3.8 × 10^5^ particles/µL). (B) Immunofluorescence staining of iNOS and Arg‐1 in LPS‐activated microglia after different treatments (Peptide‐Apt_CD63_, 37.4 µm; MSC‐Exo, 3.8 × 10^5^ particles/µL) for 24 h. (C) Microscopic images of NSCs before and after different treatments for 24 h in a cell migration assay (Peptide‐Apt_CD63_, 37.4 µm; MSC‐Exo, 3.8 × 10^5^ particles/µL). Relative mean fluorescence intensity (MFI) of (D) immunostained iNOS and (E) Arg‐1 in LPS‐activated microglia after different treatments for 24 h in the immunofluorescence images. Relative mRNA expression levels of (F) iNOS and (G) Arg‐1 in LPS‐activated microglia after different treatments for 24 h. (H) Statistical analysis of migration ratios of NSCs in different groups at 24 h post‐treatment. (I) Proliferation of NSCs after treatment with Peptide‐Apt_CD63_/Exo microgels at different MSC‐Exo concentrations for 24 h. All data are represented by mean ± SD (n = 3, ^*^ *p* < 0.05, ^**^ *p* < 0.01, ^***^ *p* < 0.001, ^****^ *p* < 0.0001).

Time‐lapse confocal microscopy tracked endocytic routes of materials in mouse NSCs, revealing robust co‐localization between DiO‐labeled MSC‐Exo in microgels and LysoTracker Red‐stained lysosomes 3 h post‐incubation, which demonstrated endolysosomal sequestration (Figure S16). To investigate the endocytic pathway of DiO‐labeled MSC‐Exo, dynamin‐mediated endocytosis was inhibited using dynasore. Notably, dynasore pretreatment significantly attenuated DiO fluorescence in NSCs, suggesting dynamin‐dependent internalization of both MSC‐Exo and Peptide‐Apt_CD63_/Exo microgels (Figure S17). To evaluate the intracellular ROS scavenging capability, NSCs were treated with a mixture of Rosup agent and Peptide‐Apt_CD63_/Exo microgels for 30 min. The Rosup agent induced the excessive production of intracellular ROS during this period. For ROS visualization and quantification, dichloro‐dihydro‐fluorescein diacetate (DCFH‐DA) was employed as an indicator probe, which fluoresces upon reaction with intracellular ROS (Figure 2A; Figure S18). In comparison to the PBS group, both MSC‐Exo‐treated cells and Peptide‐Apt_CD63_‐treated cells exhibited notable fluorescence quenching. The fluorescence quenching effect observed in MSC‐Exo‐treated cells is likely attributable to the active regulation of cellular antioxidant signaling pathways by MSC‐Exo [30, 31, 32], whereas the decrease in fluorescence in Peptide‐Apt_CD63_‐treated cells is linked to the intrinsic antioxidant properties of thioether groups within the polypeptide [27, 28, 29]. Notably, the Peptide‐Apt_CD63_/Exo group displayed almost negligible fluorescence, indicating a significantly enhanced intracellular ROS scavenging capability of the Peptide‐Apt_CD63_/Exo microgels. This finding highlights the potential of Peptide‐Apt_CD63_/Exo microgels as a promising platform for scavenging ROS at the SCI site in vivo (see below).

It is well known that inducible NO synthase (iNOS) and arginase‐1 (Arg‐1) are the markers of M1 (pro‐inflammatory) and M2 (anti‐inflammatory) microglia [33] and macrophages [34], respectively. Therefore, the expression levels of iNOS and Arg‐1 were analyzed via immunofluorescence staining and flow cytometry to evaluate the polarization state of microglia (Figure 2B,D, and E; Figure S19) and macrophages (Figures S20–S22) after different treatments. Apparently, the fluorescence intensity of immunostained iNOS was weakened, and the fluorescence intensity of immunostained Arg‐1 was elevated by MSC‐Exo and Peptide‐Apt_CD63_ compared to PBS alone. Nevertheless, the Peptide‐Apt_CD63_/Exo group displayed the weakest fluorescence of immunostained iNOS and the most elevated fluorescence of immunostained Arg‐1. Furthermore, we revealed that the mRNA expression level of iNOS in LPS‐activated microglia was downregulated by Exo (∼0.24‐fold), Peptide‐Apt_CD63_ (∼0.29‐fold), and Peptide‐Apt_CD63_/Exo (∼0.14‐fold), whereas the mRNA expression level of Arg‐1 was upregulated by MSC‐Exo (∼2.15‐fold), Peptide‐Apt_CD63_ (∼1.13‐fold), and Peptide‐Apt_CD63_/Exo (∼2.70‐fold) respectively, as compared to the PBS group (Figure 2F,G). A similar trend was observed in LPS‐activated macrophages after treatment with Peptide‐Apt_CD63_/Exo (Figure S23). The shift in polarization phenotype was further confirmed through downregulating the mRNA expression levels of other pro‐inflammatory factors (e.g., tumor necrosis factor alpha (TNF‐α), matrix metalloproteinase 9 (MMP9)), and upregulated the mRNA expression levels of other anti‐inflammatory factors (e.g., interleukin 10 (IL‐10), transforming growth factor‐beta (TGF‐β)) (Figure S24). This phenotypic switch by MSC‐Exo was likely attributed to their roles in activating antioxidant signaling pathways (e.g., MAPK/NF‐κB, Nrf2/Keap1), as validated in the previous work [35, 36]. Meanwhile, this effect of Peptide‐Apt_CD63_ is likely ascribed to its thioether groups, which can react with ROS, thus creating a redox environment conducive to M2 polarization through ROS scavenging [37, 38]. In this context, the combination of MSC‐Exo and Peptide‐Apt_CD63_ within microgels demonstrated remarkable anti‐inflammatory effects, characterized by the most significant reduction in iNOS expression and the most significant enhancement in Arg‐1 expression.

Next, we cultured NSCs with Peptide‐Apt_CD63_/Exo microgels to investigate their migration and proliferation. As shown in Figure 2C, cell migration was moderately promoted by Peptide‐Apt_CD63_ and Exo, whereas it was significantly promoted by Peptide‐Apt_CD63_/Exo microgels. Remarkably, the cell migration ratio in the Peptide‐Apt_CD63_/Exo group was 2.24‐fold and 2.73‐fold higher than that in the Peptide‐Apt_CD63_ and Exo groups, respectively (Figure 2H). Meanwhile, the proliferation ratio of NSCs was significantly enhanced by Peptide‐Apt_CD63_/Exo microgels as a function of MSC‐Exo concentration, with an increase of ∼30% when NSCs were treated with microgels containing 4.0 × 10^5^ particles/µL MSC‐Exo (Figure 2I). These findings highlight the potential of Peptide‐Apt_CD63_/Exo microgels to accelerate SCI repair by promoting migration and proliferation of NSCs.

### 2.3 In Vitro Differentiation of NSCs into Neurons

**FIGURE 3:**
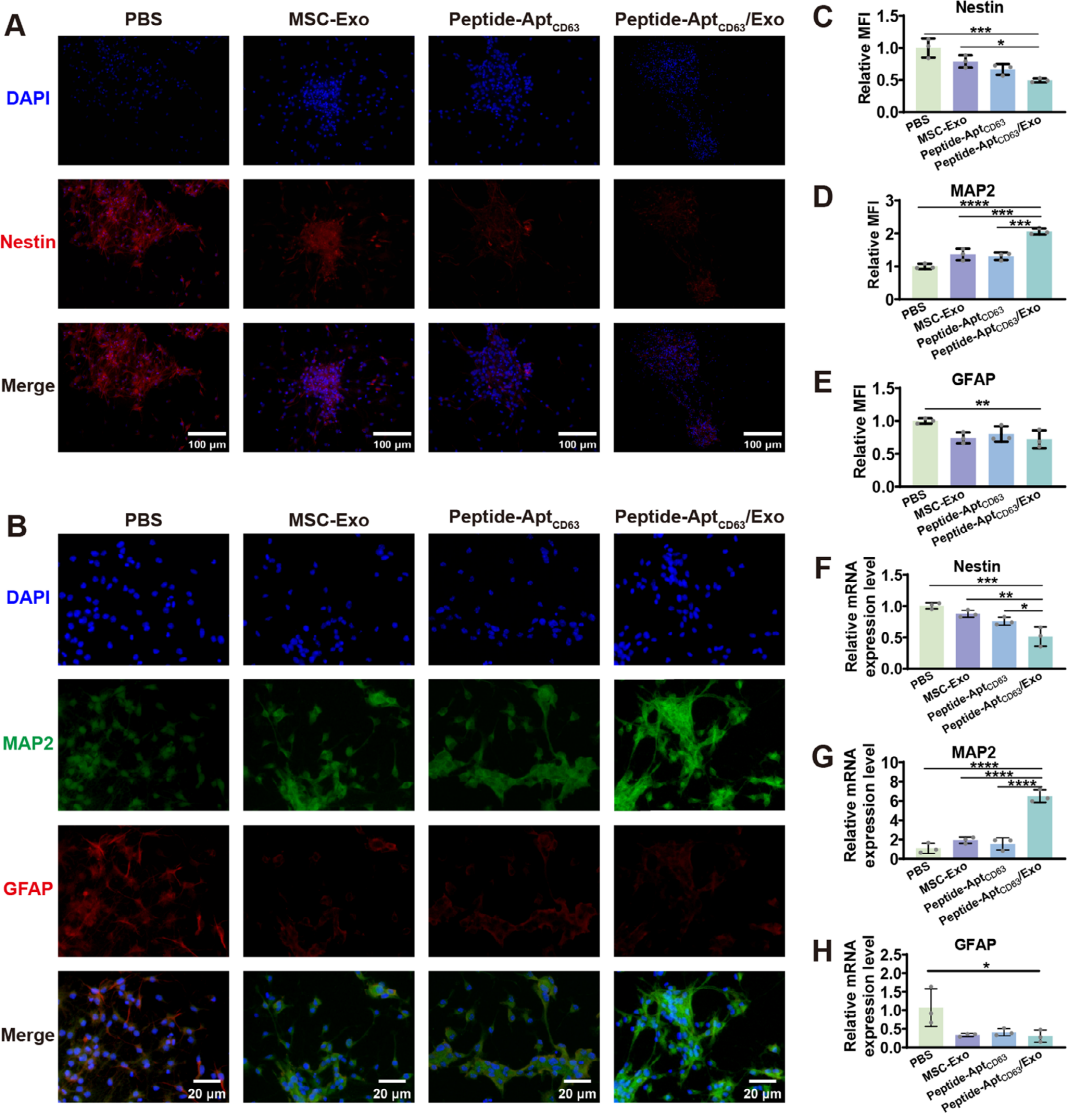
In vitro differentiation of NSCs into neurons. Immunofluorescence staining of (A) Nestin and (B) MAP2 and GFAP of NSCs after different treatments in differentiation culture medium for 7 days. Statistical analysis of MFI of (C) immunostained Nestin, (D) MAP2, and (E) GFAP in the immunofluorescence images. Relative mRNA expression levels of (F) Nestin, (G) MAP2, and (H) GFAP. All data are represented by mean ± SD (n = 3, ^*^ *p* < 0.05, ^**^ *p* < 0.01, ^***^ *p* < 0.001, ^****^ *p* < 0.0001).

Since newly formed neurons are crucial to the SCI neuroregeneration [39, 40], we hence investigated the influence of Peptide‐Apt_CD63_/Exo microgels on the differentiation of the NSCs into neurons. To this end, NSCs were subjected to different treatments in the differentiation medium for 7 days, followed by immunofluorescence staining of neuroepithelial stem cell protein (Nestin) (a NSC marker), microtubule‐associated protein 2 (MAP2) (a neuron marker), and glial fibrillary acidic protein (GFAP) (an astrocyte marker). Clearly, the NSCs in the Peptide‐Apt_CD63_/Exo group displayed the weakest immunofluorescence signals of Nestin and GFAP, but the strongest immunofluorescence signal of MAP2, compared to both the MSC‐Exo and Peptide‐Apt_CD63_ groups (Figure 3A–E). Notably, this group also presented a substantial number of MAP2‐positive cells characterized by extensive slender neurites. Furthermore, the differentiation of NSCs was assessed using RT‐qPCR. As shown in Figure 3F–H, the Peptide‐Apt_CD63_/Exo group exhibited the most pronounced increase in the mRNA expression level of MAP2 (∼6.50‐fold), alongside a marked decrease in the mRNA expression levels of Nestin (∼0.52‐fold) and GFAP (∼0.31‐fold) compared to both the MSC‐Exo (∼1.93‐fold for MAP2, ∼0.88‐fold for Nestin, and ∼0.34‐fold for GFAP) and Peptide‐Apt_CD63_ (∼1.55‐fold for MAP2, ∼0.76‐fold for Nestin, and ∼0.41‐fold for GFAP) groups. Evidently, the differentiation of NSCs were promoted synergistically by MSC‐Exo and Peptide‐Apt_CD63_, alongside enhanced biostability of Exo conferred by the gel matrix [41, 42]. The underlying mechanisms for the increased MAP2 expression and decreased Nestin/GFAP expression involve MSC‐Exo cargos (e.g., neurotrophic factors [43, 44] and miRNAs [45, 46, 47]) activating MAPK and PI3K‐Akt signaling pathways, coupled with glutamic acid liberation upon Peptide‐Apt_CD63_ degradation that triggers intracellular Ca^2+^‐dependent signaling [48, 49]. These activated pathways collectively promote neuronal commitment and maturation, thereby accounting for the observed synergistic effects. Collectively, these results indicate that Peptide‐Apt_CD63_/Exo microgels significantly fostered the differentiation of NSCs towards a neuronal fate rather than astrocytic lineage, underscoring their potential to facilitate SCI repair and the reconstruction of neural circuits.

### 2.4 Motor Function Recovery of SCI Mice

**FIGURE 4:**
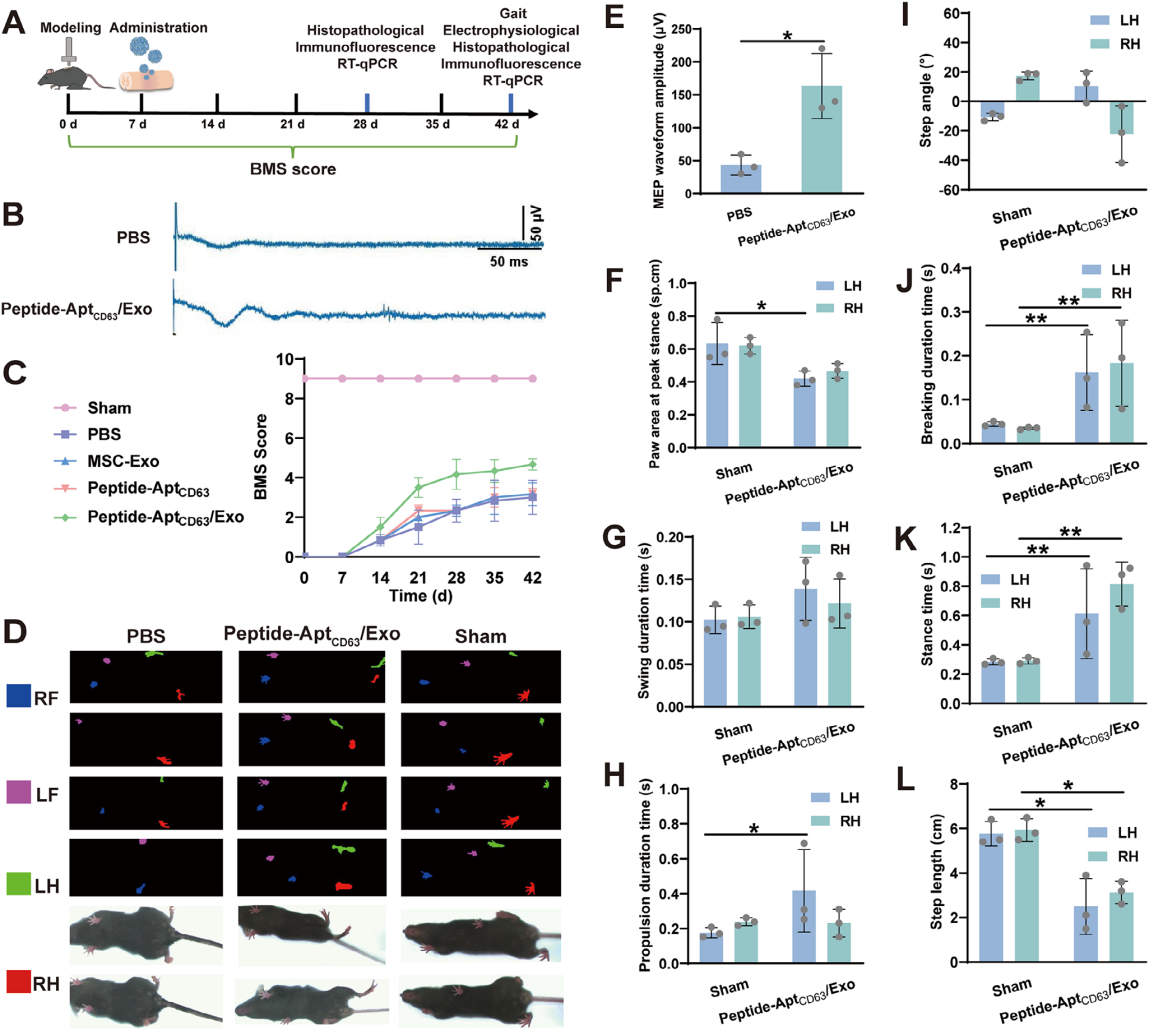
Motor function recovery of SCI mice. (A) Schematic illustration of the establishment and treatments of SCI model. (B) Electrophysiological signal analysis of SCI mice treated with PBS and Peptide‐Apt_CD63_/Exo microgels at 6 weeks post‐surgery. (C) BMS score profiles of bilateral hindlimbs for sham mice and SCI mice after different treatments from 0 weeks to 6 weeks post‐surgery. (D) Footprinting of mice in the sham group and the Peptide‐Apt_CD63_/Exo group at 6 weeks post‐surgery. LF: left front; RF: right front; LH: left hind; RH: right hind. (E) Motor‐evoked potential (MEP) waveform amplitude of SCI mice after treated with PBS and Peptide‐Apt_CD63_/Exo microgels at 6 weeks post‐surgery (n = 3). (F) Paw area at peak stance, (G) swing duration time, (H) propulsion duration time, (I) step angle, (J) breaking duration time, (K) stance time, and (L) step length of mice in the sham group and the Peptide‐Apt_CD63_/Exo group, respectively, at 6 weeks post‐surgery (n = 3). All data are represented by mean ± SD (n = 3, ^*^ *p* < 0.05, ^**^ *p* < 0.01, ^***^ *p* < 0.001, ^****^ *p* < 0.0001).

Given their verified superior bioactivities, excellent cytocompatibility, and negligible apoptosis, necrosis, and hemolysis (Figures S25–S27), Peptide‐Apt_CD63_/Exo microgels were utilized to promote neural circuit formation in SCI mice. To this end, the mice were subjected to spinal T10 segment percussion surgery after laminectomy (day 0), and the microgels were administered on day 7 (Figure 4A). Thereafter, motor‐evoked potentials (MEPs) from the hindlimbs were recorded following electrical stimulation of the brain at 6 weeks post‐surgery. Clearly, SCI mice treated with Peptide‐Apt_CD63_/Exo microgels (mean MEP amplitude 163.3 µV) exhibited significantly enhanced motor‐evoked responses compared to those treated with PBS (mean amplitude 43.3 µV) (Figure 4B,E). In addition, motor function recovery was evaluated using the Basso Mouse Scale (BMS) open‐field score over a 6‐week period (Figure 4C; Table S1). Following the strike injury, SCI mice exhibited complete paralysis of the hind limbs, reflected by a BMS score of 0. In contrast, sham mice exhibited unobvious motor deficits, with a mean BMS score of 9.0, as laminectomy did not impact the spinal cord or locomotion. Over a 6‐week recovery period, the Peptide‐Apt_CD63_/Exo group demonstrated consistently higher BMS scores compared to the PBS, MSC‐Exo, and Peptide‐Apt_CD63_ groups. At 6 weeks post‐surgery, SCI mice in the Peptide‐Apt_CD63_/Exo group achieved a BMS score of 4.7, indicating consistent dorsal stepping with plantar support, while the SCI mice in the other groups remained below 3.0, reflecting only occasional ankle movement without weight‐supported stepping.

Furthermore, gait analyses were conducted to assess the recovery of motor behavior of mice at 42 days post‐SCI (Figure 4D). Clearly, mice in the Sham group exhibited normal locomotion with consistent plantar stepping, regular stride intervals, and stable weight‐bearing during stance phases. In contrast, SCI mice in the PBS group showed significantly impaired gait characterized by ataxic movements (e.g., limb dragging or lateral instability). SCI mice receiving Peptide‐Apt_CD63_/Exo treatment demonstrated obvious functional recovery, evidenced by restored plantar stepping consistency and improved inter‐limb coordination, though residual deficits persisted (e.g., paw rotation during swing phases). Since the complete loss of hindlimb function in the PBS group precluded the acquisition of valid gait parameters, comparative statistical gait analysis focused on the Peptide‐Apt_CD63_/Exo and Sham groups. This analysis revealed that Peptide‐Apt_CD63_/Exo significantly restored weight‐bearing capacity, evidenced by recovered paw contact area at peak stance phase to sham‐comparable levels (Figure 4F). Markedly improved interlimb coordination in the Peptide‐Apt_CD63_/Exo group was observed, with swing phase duration approaching sham group values (Figure 4G). Propulsion phase duration of the right hindlimb in the Peptide‐Apt_CD63_/Exo group showed significant recovery (Figure 4H), indicating effective restoration of ipsilateral motor control. Asymmetric paw rotation was comparable between the Sham group and the Peptide‐Apt_CD63_/Exo group (Figure 4I), suggesting improved motor coordination. The presence of breaking phase duration (Figure 4J), stance phase duration (Figure 4K), and step length (Figure 4L)—parameters absent in PBS group – collectively demonstrates regained locomotor capability, aligning with stability‐focused gait restoration in SCI [50, 51, 52]. Taken together, these findings demonstrate that Peptide‐Apt_CD63_/Exo microgels significantly enhanced the functional recovery of SCI mice, with several key motor metrics approaching to the sham group.

### 2.5 In Vivo Therapeutic Mechanisms of SCI Lesions

**FIGURE 5:**
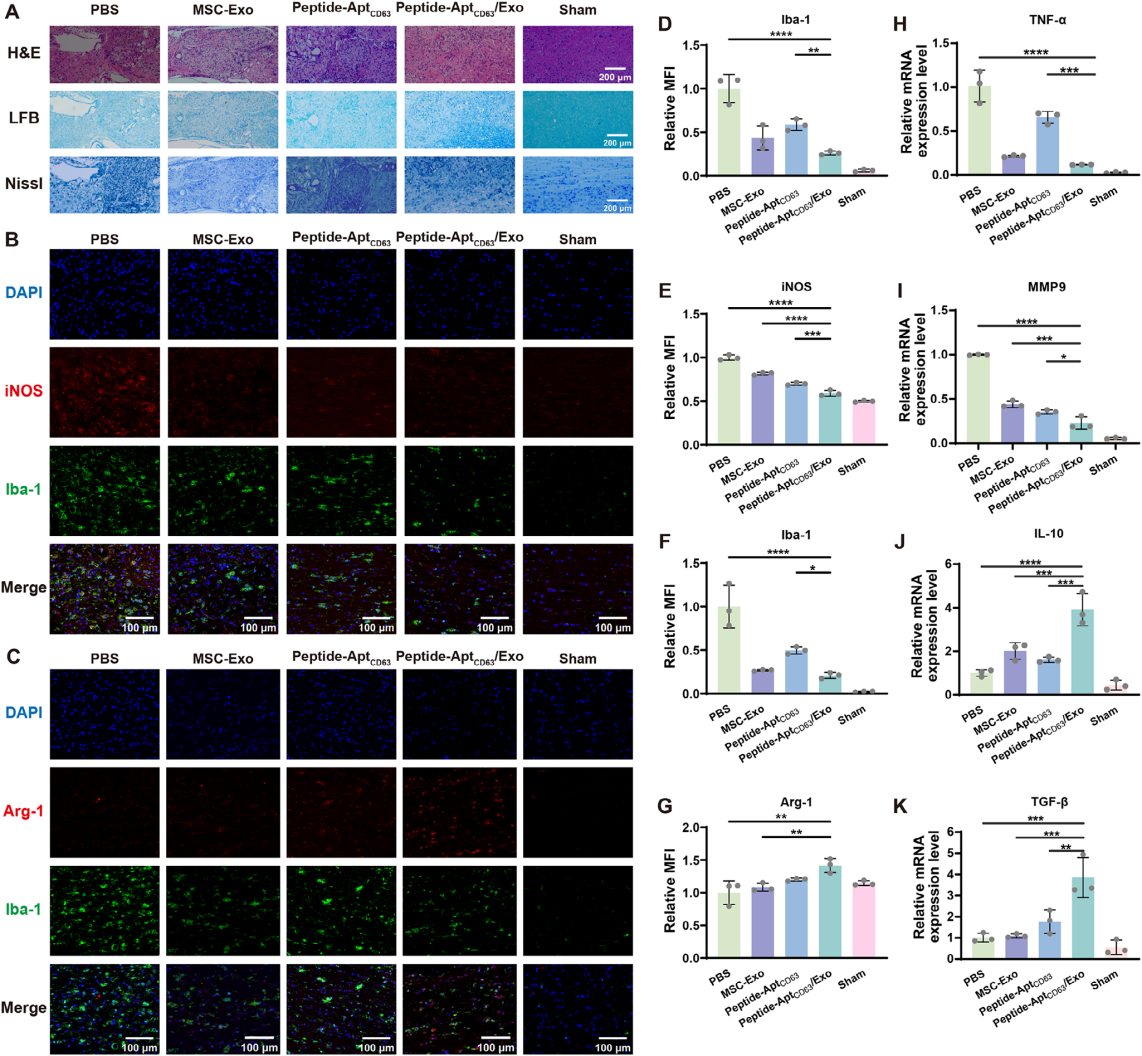
In vivo histological recovery and immunomodulation. (A) H&E, LFB, and Nissl staining of the injury sites in different groups at 6 weeks post‐surgery. Immunofluorescence staining of (B) Iba‐1/iNOS and (C) Iba‐1/Arg‐1 of the injury sites in different groups at 6 weeks post‐surgery. Relative MFI of (D) immunostained Iba‐1, (E) iNOS, (F) Iba‐1, and (G) Arg‐1 in the immunofluorescence images. Relative mRNA expression levels of (H) TNF‐α, (I) MMP9, (J) IL‐10, and (K) TGF‐β of the injury sites in different groups at 6 weeks post‐surgery. All data are represented by mean ± SD (n = 3, ^*^ *p* < 0.05, ^**^ *p* < 0.01, ^***^ *p* < 0.001, ^****^ *p* < 0.0001).

Next, we investigated the mechanisms of SCI repair process mediated by Peptide‐Apt_CD63_/Exo microgels through histological examinations. Hematoxylin and eosin (H&E) staining revealed notable atrophy and vacuolation of the lesion site, accompanied by a large number of inflammatory cell infiltrations in the PBS group at 6 weeks post‐surgery (Figure 5A). However, those pathological features were markedly alleviated to the most extent in the Peptide‐Apt_CD63_/Exo group, compared to the MSC‐Exo and Peptide‐Apt_CD63_ groups. Meanwhile, Luxol Fast Blue (LFB) staining was employed to assess demyelination in the injured spinal cord tissue [53, 54, 55], with staining intensity positively correlating with myelin content. Apparently, the Peptide‐Apt_CD63_/Exo group exhibited the most intense staining, indicating superior myelin regeneration relative to the other groups. Meanwhile, Nissl bodies were stained due to their critical roles in protein synthesis and synaptic plasticity within neurons [56]. Similarly, the obvious presence of Nissl bodies was prominently observed only in the Peptide‐Apt_CD63_/Exo group, further validating the robust recovery of spinal neurons facilitated by the microgels. To strengthen the conclusion, similar histological examinations were conducted at 4 weeks post‐surgery (Figures S28–S30).

Following SCI, microglia, the primary immune sentinels in the central nervous system, are typically activated into the M1 phenotype [57]. Conversely, transitioning microglia from the M1 to the M2 phenotype promotes SCI repair [58, 59]. At 6 weeks post‐surgery, Peptide‐Apt_CD63_/Exo treatment significantly reduced the expression of Iba‐1/iNOS, markers for M1 microglia (Figure 5B,D, and E), while it simultaneously elevated the expression of Arg‐1, a marker for M2 microglia (Figure 5C,F, and G), compared to other treatments. This suggests a significant reduction of microglia and conversion of the M1 to the M2 microglia by Peptide‐Apt_CD63_/Exo microgels. Furthermore, we analyzed the mRNA expression levels of downstream pro‐inflammatory factors, including TNF‐α and MMP9 [60, 61], as well as anti‐inflammatory factors such as IL‐10 and TGF‐β [62, 63] using RT‐qPCR. These results indicated that Peptide‐Apt_CD63_/Exo microgels markedly downregulated the expression levels of pro‐inflammatory TNF‐α (∼0.12‐fold) and MMP9 (∼0.23‐fold) and simultaneously upregulated anti‐inflammatory factors like IL‐10 (∼3.91‐fold) and TGF‐β (∼3.86‐fold) as compared to MSC‐Exo (TNF‐α, ∼0.22‐fold; MMP9, ∼0.44‐fold; IL‐10, ∼2.01‐fold; TGF‐β, ∼1.09‐fold) and Peptide‐Apt_CD63_ (TNF‐α, ∼0.66‐fold; MMP9, ∼0.35‐fold; IL‐10, ∼1.61‐fold; TGF‐β, ∼1.77‐fold) (Figure 5H–K). This enhanced immunomodulatory efficacy of Peptide‐Apt_CD63_/Exo microgels indicates a superior capacity to reprogram the SCI microenvironment toward a repair‐conducive state. To strengthen the conclusion, similar histological (Figures S31–S34) and RT‐qPCR analyses (Figures S35 and S36) were conducted at 4 weeks post‐surgery.

**FIGURE 6:**
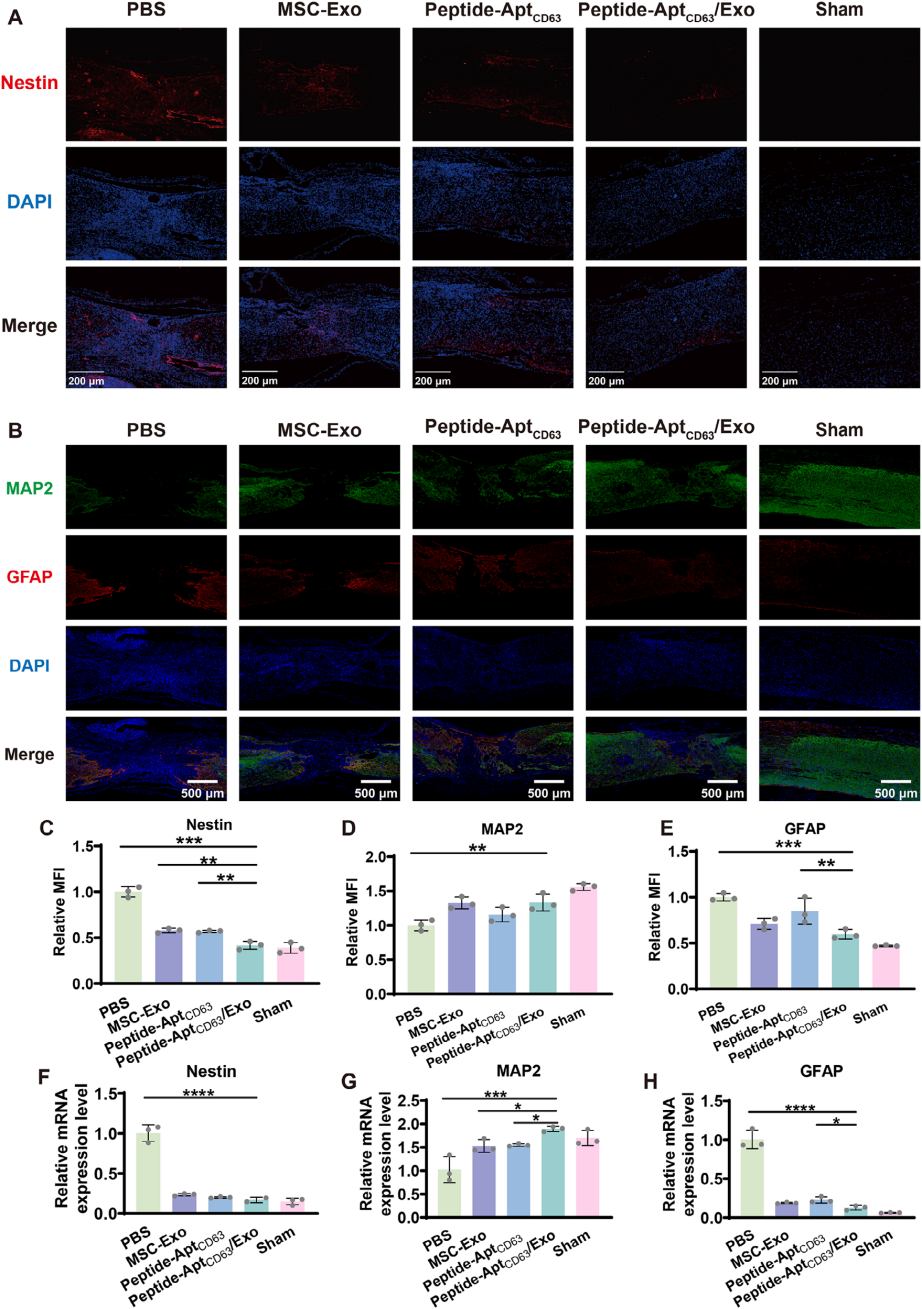
In vivo neuroregeneration. (A) Immunofluorescence staining of Nestin of the injury sites in different groups at 6 weeks post‐surgery. (B) Immunofluorescence staining of MAP2 and GFAP of the injury sites in different groups at 6 weeks post‐surgery. Relative MFI of (C) Nestin, (D) MAP2, and (E) GFAP in the immunofluorescence images. Relative mRNA expression levels of (F) Nestin, (G) MAP2, and (H) GFAP of the injury sites in different groups at 6 weeks post‐surgery. All data are represented by mean ± SD (n = 3, ^*^ *p* < 0.05, ^**^ *p* < 0.01, ^***^ *p* < 0.001, ^****^ *p* < 0.0001).

Next, we conducted immunofluorescence staining to analyze the expression levels of Nestin, MPA2, and GFAP, providing insights into the differentiation trajectories of NSCs (Figure 6; Figures S37–S41). Evidently, the Peptide‐Apt_CD63_/Exo group displayed the weakest immunofluorescence signals of Nestin and GFAP, but the strongest immunofluorescence signal of MAP2, compared to the MSC‐Exo and Peptide‐Apt_CD63_ groups at both 4 and 6 weeks post‐surgery. These results suggest that Peptide‐Apt_CD63_/Exo microgels significantly fostered the differentiation of NSCs towards a neuronal fate rather than astrocytic lineage. Furthermore, the differentiation of NSCs in the lesions was assessed using RT‐qPCR (Figure 6F–H; Figures S39, and S42). While the expression of Nestin at the injury site was reduced by both MSC‐Exo (∼0.92‐fold at 4 weeks post‐surgery; ∼0.24‐fold at 6 weeks post‐surgery) and Peptide‐Apt_CD63_ (∼0.99‐fold at 4 weeks post‐surgery; ∼0.20‐fold at 6 weeks post‐surgery) treatments, it was lower following the Peptide‐Apt_CD63_/Exo treatment (∼0.40‐fold at 4 weeks post‐surgery; ∼0.17‐fold at 6 weeks post‐surgery) compared to the PBS treatment. This reduction is attributed to the ability of Peptide‐Apt_CD63_/Exo microgels to effectively facilitate the differentiation of NSCs into mature neurons (Figure 3). This conclusion is further supported by the increased expression of MAP2 at the injury sites. Apparently, the highest expression level of MAP2 at the injury site was observed in the Peptide‐Apt_CD63_/Exo group (∼2.31‐fold at 4 weeks post‐surgery; ∼1.89‐fold at 6 weeks post‐surgery), compared to other groups with SCI mice (MSC‐Exo: ∼2.06‐fold at 4 weeks post‐surgery, ∼1.53‐fold at 6 weeks post‐surgery; Peptide‐Apt_CD63_: ∼1.61‐fold at 4 weeks post‐surgery, ∼1.55‐fold at 6 weeks post‐surgery). Notably, GFAP expression was significantly reduced in the Peptide‐Apt_CD63_/Exo group (∼0.30‐fold at 4 weeks post‐surgery; ∼0.13‐fold at 6 weeks post‐surgery) relative to other groups with SCI mice (MSC‐Exo: ∼0.55‐fold at 4 weeks post‐surgery, ∼0.19‐fold at 6 weeks post‐surgery; Peptide‐Apt_CD63_: ∼0.51‐fold at 4 weeks post‐surgery, ∼0.23‐fold at 6 weeks post‐surgery), which is crucial for reducing glial scar formation and thereby potentially facilitating a microenvironment more conducive to axonal regeneration [64]. These findings suggest that Peptide‐Apt_CD63_/Exo microgels promoted neuroregeneration through facilitating the differentiation of NSCs into neurons rather than astrocytes. Consequently, the SCI mice in the Peptide‐Apt_CD63_/Exo group established a more complete neural circuit, facilitating enhanced signal transmission, which aligns with the results from the MEP assessments (Figure 4). H&E staining of major organs revealed no significant pathological lesions in mice injected with Peptide‐Apt_CD63_/Exo microgels compared to PBS controls seven days post‐injection (Figure S43), indicating the material's in vivo safety.

## 3 Conclusion

In summary, we developed an unprecedented assembly strategy that leveraged immunoaffinity‐mimetic interactions, with Peptide‐Apt_CD63_ conjugates acting as “antibody” surrogates binding CD63 “epitopes” on MSC‐Exo, creating a hierarchical microstructure designed to promote SCI repair. The Peptide‐Apt_CD63_ conjugate was synthesized via ROP of PRC‐NCA and BLG‐NCA monomers, followed by the grafting of an azide‐modified Apt_CD63_. Given the multivalent Apt_CD63_ ligands on each conjugate and abundant CD63 receptors on each MSC‐Exo, their specific interactions resulted in the formation of a 3D crosslinked architecture, designated as the Peptide‐Apt_CD63_/Exo microgel. Upon implantation of the microgel into the injury site of a SCI mouse model, the presence of thioether groups within the polypeptide facilitated the effective clearance of ROS, thereby alleviating the oxidative stress and the inflammatory responses. The synergistic anti‐inflammatory effect of Peptide‐Apt_CD63_ conjugate in conjunction with MSC‐Exo significantly facilitated the transition of M1‐type microglia to the M2 phenotype. This transformation was accompanied by a marked downregulation of mRNA expression levels of pro‐inflammatory TNF‐α (∼0.12‐fold) and MMP9 (∼0.23‐fold), and a notable upregulation of mRNA expression levels of anti‐inflammatory factors like IL‐10 (∼3.91‐fold) and TGF‐β (∼3.86‐fold) at 6 weeks post‐surgery. This alteration in the microenvironment is conducive to the proliferation and migration of NSCs to the injury site and their differentiation into neurons [65]. Furthermore, the proliferation (∼1.30‐fold) and migration (∼5.43‐fold) of NSCs were significantly promoted after in vitro cultivation with Peptide‐Apt_CD63_/Exo microgels. Importantly, Peptide‐Apt_CD63_/Exo microgels downregulated the mRNA expression level of Nestin (∼0.40‐fold at 4 weeks post‐surgery; ∼0.17‐fold at 6 weeks post‐surgery) and upregulated the mRNA expression level of MAP2 (∼2.31‐fold at 4 weeks post‐surgery; ∼1.89‐fold at 6 weeks post‐surgery). Meanwhile, the microgels significantly reduced the mRNA expression level of GFAP (∼0.30‐fold at 4 weeks post‐surgery; ∼0.13‐fold at 6 weeks post‐surgery). Therefore, the microgels facilitated the differentiation of NSCs into neurons and suppressed astrogliosis associated with glial scar formation. This favorable shift in cell fate was further supported by morphological evidence of neuronal health (high Nissl body content via Nissl staining) and enhanced myelination (via LFB staining), suggesting their potential in promoting neuroregeneration. Ultimately, the characteristics of the atrophy and vacuolation of the lesion site, along with a large number of inflammatory cell infiltrations, were reversed, leading to a significant recovery of motor function in the SCI mice after treatment with microgels. A central theme of this study is the immunoaffinity‐mimetic assembly of oligonucleotide conjugates and biologics. We anticipate that a diverse array of scale‐spanning materials with synergistic physicochemical properties can be customized through the precise tailoring of oligonucleotide sequences and the incorporation of various vulnerable biologics for novel biomedical applications beyond SCI repair [66, 67, 68], thereby providing a new perspective for broad applications across materials science and life science.

## 4 Experimental Section/Methods

### 4.1 Assembly of Peptide‐Apt_CD63_/Exo Microgels

Peptide‐Apt_CD63_ conjugate with alkynyl groups was synthesized via the ROP of PRC‐NCA and BLG‐NCA monomers, followed by the grafting of an azido group‐modified Apt_CD63_. A full description of experimental procedures can be found. Then, Peptide‐Apt_CD63_ (25–150 µm Apt_CD63_) was mixed with MSC‐Exo (5.0 × 10^5^ particles/µL) in 20 µL of PBS, and the mixture was incubated at 4 °C for 20 h. Excess reagents in the supernatant were then carefully removed to yield Peptide‐Apt_CD63_/Exo microgels. A full description of materials characterization can be found.

### 4.2 Biological Activity In Vitro

Fluorescence microscopy, RT‐qPCR, and other methods were used to study the biofunctionalities of microgels in vitro, such as antioxidation, immunomodulation, NSC proliferation, NSC migration, and NSC differentiation. A full description of experimental procedures can be found.

### 4.3 Therapeutic Efficacy In Vivo

All animal experiments were approved by the Institutional Animal Care and Use Committee at Tongji University (approval # TJAF00125102) and carried out according to protocols approved by the committee for animal care and in accordance with the National Ministry of Health. In vivo therapeutic efficacy of Peptide‐Apt_CD63_/Exo microgels was evaluated by establishing a strike SCI model and conducting behavioral assessment, gait analysis, and electrophysiological analysis. To further reveal the therapeutic mechanism, the spinal cord tissue structure, axonal remyelination, Nissl body density, pro‐regenerative immunomodulation, and differentiation of NSCs at the injury sites were analyzed by histopathological staining, immunofluorescence staining, and RT‐qPCR. A full description of experimental procedures can be found.

### 4.4 Statistical Analysis

Data pre‐processing included transformation and normalization. Statistical analysis was conducted using Graphpad Prism 8.0 software. All values are presented as the mean ± SD. A sample size of n ≥ 3 was used for each statistical analysis. One‐way ANOVA was used for multiple comparisons of the groups, and Student's t‐test was used to determine the significance of differences between two groups. A significance level of *p* < 0.05 was considered statistically significant (^*^ *p* < 0.05, ^**^ *p* < 0.01, ^***^ *p* < 0.001, ^****^ *p* < 0.0001).

## Funding

This research was supported by the National Natural Science Foundation of China (52373154), the Shanghai Rising Star Program (2023QA1411700), the Interdisciplinary Project in “Medicine + X” of Tongji University, and the Fundamental Research Funds for the Central Universities.

## Conflicts of Interest

The authors declare no conflicts of interest.

## Supporting information




**Supporting File**: advs73706‐sup‐0001‐SuppMat.docx.

## Data Availability

The data that support the findings of this study are available from the corresponding author upon reasonable request.
