# Supplementary material for: Immunoaffinity‐Mimetic Assembly of Peptide‐Aptamer Conjugates and Stem Cell‐Derived Exosomes into Hierarchical Microgels for Spinal Cord Injury Repair
Source: Adv Sci (Weinh). 2026 Jan 9;13(16):e19701. doi: 10.1002/advs.202519701 (PMC13042431; doi:10.1002/advs.202519701)
Supplement: Supplementary file 1 — Supporting File: advs73706‐sup‐0001‐SuppMat.docx. [file ADVS-13-e19701-s001.docx]

Immunoaffinity-Mimetic Assembly of Peptide-Aptamer Conjugates and Stem Cell-Derived Exosomes into Hierarchical Microgels for Spinal Cord Injury Repair

Dantong Zheng, Yuxin Bai, Zhibo Liu, Zinan Zhao, Yiming Kong, Yao Xu, Qi Jia*, Rongrong Zhu*, Yong Hu*

D. Zheng, Z. Zhao, Y. Kong, Y. Xu, Prof. Dr. Y. Hu

Department of Polymeric Materials, School of Materials Science and Engineering, Tongji University, Shanghai 201804, China

E-mail: yonghu@tongji.edu.cn

Dr. Y. Bai, Z. Liu, Prof. R. Zhu

Key Laboratory of Spine and Spinal Cord Injury Repair and Regeneration of Ministry of Education, Department of Orthopedics, School of Life Sciences and Technology, Tongji Hospital affiliated to Tongji University, Tongji University, Shanghai 200065, China

E-mail: rrzhu@tongji.edu.cn

Prof. Dr. Q. Jia

Department of Orthopedic Oncology, Shanghai Changzheng Hospital, Second Affiliated Hospital of Naval Medical University, Shanghai 201209, China

E-mail: qj1215@smmu.edu.cn

1. Experimental Section

1.1. Materials

Propargyl cysteine (PRC), γ-benzyl-L-glutamate N-carboxyanhydride (BLG-NCA), n-hexylamine, and dynasore were purchased from Innochem Technology Co., Ltd. (Tianjin, China). Phosphate buffer saline (PBS), N, N-Dimethylformamide (DMF), normal saline, and tetrahydrofuran (THF) were purchased from Aladdin (Shanghai, China). Milk exosomes (Milk-Exo, batch number # 45110156) was purchased from Umibio Co., Ltd. (Shanghai, China). MSC exosomes (MSC-Exo, batch number # 20231131) were purchased from Dlmbiotech Co., Ltd. (Wuhan, China). Oligonucleatides and DNase I were purchased from Sangon Biotech Co., Ltd. (Shanghai, China). Lipopolysaccharide (LPS), 4',6-diamidino-2-phenylindole (DAPI), Cell Count Kit 8 (CCK-8), Total Antioxidant Capacity Assay Kit with ABTS method (T-AOC Assay Kit), Reactive Oxygen Species Assay Kit, Calcein AM/propidium iodide (PI) Cell Viability/Cytotoxicity Assay Kit, and DiO (cell membrane green fluorescent probe) were purchased from Beyotime Co., Ltd. (Shanghai, China). LysoTracker Red was purchased from KeyGEN BioTECH Co., Ltd. (Nanjing, China). Hoechst 33342 was purchased from Solarbio Science & Technology Co., Ltd. (Beijing, China). Cluster of differentiation 63 (CD63) ELISA Kit was pruchased from CusaBio Co., Ltd. (Wuhan, China). Recombinant Mouse EGF (C-6His) and recombinant Mouse bFGF were purchased from Novoprotein (Suzhou, China). B-27 and N-2 serum-free additive were purchased from Zhong Qiao Xin Zhou Biotechnology Co., Ltd. (Shanghai, China). Human umbilical vein endothelial cells (HUVECs, Catalog # TCH-C406-UD), was purchased from Cas9X (Suzhou, China). Mouse macrophages (RAW 264.7, Catalog # TCM-C766) were purchased from Hysigen Bioscience Co., Ltd. (Shanghai China). Mouse microglia (SIM-A9, Catalog # YS250682) were purchased from Shanghai Yaji Biotechnology Co., Ltd. (Shanghai, China). Dulbecco's Modified Eagle Medium (DMEM) high glucose medium, DMEM/F-12 medium, penicillin/streptomycin and fetal bovine serum (FBS) were purchased from Gibco (Grand Island, NY). All other chemicals were available from commercial sources and used without further purification.

1.2. Synthesis of PRC-NCA

To synthesize PRC-NCA monomer, 0.50 g of PRC and 0.49 g of triphosgene were added to a round-bottom flask, followed by the addition of 1.43 mL of α-pinene and 10 mL of anhydrous DMF. The mixture was stirred at 400 rpm, 55 °C for 5 h in a glove box. Once the reaction mixture clarified, it was cooled to room temperature. The crude NCA monomer was precipitated three times in pre-chilled n-hexane under stirring. The precipitate obtained after centrifugation was vacuum-dried to yield PRC-NCA monomer.

1.3. Synthesis of Peptide-Apt_CD63_ conjugate

0.28 g of PRC-NCA and 1.31 g of BLG-NCA were dissolved in 50 mL of anhydrous DMF in a round-bottom flask. A tenfold dilution of n-hexylamine was prepared using anhydrous DMF, and 43.5 µL of this solution was injected in the round-bottom flask, stirring at 150 rpm for 72 h in a glove box. The reaction solution was then poured into a large excess of anhydrous ether to precipitate the product. The resulting product was collected by centrifugation, washed with ether, and dried under vacuum. The molecular weight of the peptide chains was measured using GPC, while the proportion of PRC in the peptide was quantified using ^1^H NMR to calculate the number of alkyne groups per peptide chain.

For the conjugation process, 50 µL of a tris-hydroxypropyltriazolylmethylamine (THPTA) solution (100 mM) was pre-mixed with 25 µL of CuSO_4_ solution (100 mM) to form a complex solution over 15 min. Polypeptides were dissolved in DMSO at a concentration of 1 mg/mL. A total of 618 µL of the polypeptide solution was mixed with 800 µL of DMSO, followed by the addition of 1200 µL of the Apt_CD63_ (5’-CAC CCC ACC TCG CTC CCG TGA CAC TAA TGC TAT TTTTTT-azido-3’) solution dissolved in 20% acetonitrile (1.2 µg/µL). The previously mixed complex solution was then added, followed by a quick addition of 25 µL of sodium ascorbate solution (1 M) to facilitate the reaction. The mixture was then subjected to mixing at 700 rpm and 45 °C for 5 h using a thermostatic mixer. Afterward, the product solution was subjected to ultrafiltration using a 30 kDa cutoff membrane, followed by dialysis for 12 h using a 3500 Da dialysis bag in deionized water. The content of free DNA in the solution was measured using a microspectrophotometer (NanoOne, Yooning Instrument, Hangzhou, China), allowing for the calculation of the number of side chain aptamers per polypeptide chain before and after the reaction. The microgels were collected for lyophilization prior to gold coating. Then, scanning electron microscopy (SEM, ZEISS GeminiSEM 300, Oberkochen, Germany) was performed to analyze the morphology of the dried samples.

1.4. Assembly of Peptide-Apt_CD63_/Exo microgels

Peptide-Apt_CD63_ conjugate with alkynyl groups was synthesized via the ROP of PRC-NCA and BLG-NCA monomers, followed by the grafting of an azido group-modified Apt_CD63_. A full description of experimental procedures can be found in Supporting Information. Then, Peptide-Apt_CD63_ (25-150 μM Apt_CD63_) was mixed with MSC-Exo (5.0×10^5^ particles/µL) or Milk-Exo (9.0×10^8^ particles/μL) in 20 µL of PBS, and the mixture was incubated at 4 °C for 20 h. Excess reagents in the supernatant were then carefully removed to yield Peptide-Apt_CD63_/Exo microgels.

1.5. Assembly efficiency determination

To determined assembly efficiency, Peptide-Apt_CD63_ and MSC-Exo were stained with SYBR Green II and CM-Dil, respectively. The fluorescence intensity was measured with a fluorescence microplate reader (TECAN Infinite F Nano, Männedorf, Switzerland) to establish a standard curve correlating fluorescence intensity with concentration. Fluorescence intensity in the supernatant before and after co-incubation for different times was measured to calculate the utilization rates of both aptamers and exosomes. Meanwhile, fluorescence microscopy (Olympus TH4-200, Tokyo, Japan) was used to analyze the co-localization of Peptide-Apt_CD63_ and MSC-Exo within the microgels. ImageJ was used to estiamte microgels size.

**1.6.** **Reactive oxygen species (ROS) scavenging**

The ROS scavenging ability of Peptide-Apt_CD63_ and Peptide-Apt_CD63_/Exo was tested by T-AOC Assay Kit. A 200 μL aliquot of ABTS working solution was added to each well of a 96-well plate. In the blank control wells, 10 μL of PBS was added. For the sample wells, 10 μL of test samples (Peptide-Apt_CD63_, 50-125 μM) was added, followed by gentle mixing. The plate was incubated at room temperature for 2-6 min, and absorbance was measured at 734 nm.

**1.7.** **Release kinetics of MSC-Exo**

The Peptide-Apt_CD63_/Exo microgel (37.4 µM Peptide-Apt_CD63_, 3.8×10^5^ particles/μL MSC-Exo) were incubated with normal saline, FBS (10%), and DNase I (5 U/mL) at 37 °C and 200 rpm in a metal bath, respectively. Then, supernatants were collected at designated time points and stained with CM-Dil. The fluorescence intensity was measured with a fluorescence microplate reader (TECAN Infinite F Nano, Männedorf, Switzerland) to calculate the cumulative release rate of MSC-Exo. The Exo morphology was photographed by transmission electron microscopy (TEM, JEOL JEM-2100Plus, Japan). The CD63 expression level on Exo was analyzed by ELISA Kit according to the instruction of the Kit.

**1.8.** **Routine cell culture**

The neural stem cells (NSCs) used in this study were isolated from the spinal cords of fetal mice. NSCs were cultured to a previously reported protocol.^[69]^ Briefly, NSCs were cultured in DMEM/F-12 medium, supplemented with 2% B-27, 1% N2, 1% penicillin/streptomycin, basic fibroblast growth factor (bFGF) (20 ng/mL), and epidermal growth factor (EGF) (20 ng/mL). As recommended by the American Type Culture Collection (ATCC), HUVECs and RAW 264.7 cells were routinely cultured in DMEM media supplemented with 10% FBS and 1% penicillin/streptomycin. Microglia cells were routinely cultured in Ham’s F-12 media supplemented with 10% FBS and 1% penicillin/streptomycin. All cell lines were verified to be free of mycoplasma, and cultured in a humidified incubator at 37 °C under 5% CO_2_.

**1.9. Cellular internalization**

To investigate the cellular uptake, MSC-Exo and Peptide-Apt_CD63_/Exo microgels were pre-labeled with the lipophilic dye DiO prior to addition to incubation with NSCs in 6-well plates (2.0×10^7^ cells per well). After co-incubation, cells were harvested and subjected to confocal microscopy and flow cytometry analysis. To inhibit clathrin-mediated endocytosis, NSCs were treated with 25 µg/mL dynasore for 1 h prior to exposure to MSC-Exo or Peptide-Apt_CD63_/Exo microgels. For fluorescent imaging, NSCs (1.0×10^6^ cells per dish) were seeded in confocal dishes. Then, DiO-labled MSC-Exo and Peptide-Apt_CD63_/Exo microgels (37.4 µM Peptide-Apt_CD63_, 3.8×10^5^ particles/μL MSC-Exo) were incubated with cells for 1, 2, and 3 h at 37 °C, respectively. Following removal of the cell culture medium, the pre-warmed LysoTracker Red staining solution was applied to the cells and incubated at 37 °C for 20 min. Then, the staining solution was subsequently replaced with fresh culture medium. Finally, nuclear counterstaining was performed using Hoechst 33342.

1.10. Intracellular anti-oxidation

Mouse neural stem cells (NSCs) were passaged every five days. On the third day after passage, cells in 1 mL DMEM/F-12 were seeded into a 12-well plate coated with poly-L-ornithine (PLO) for adherent culture. A dichloro-dihydro-fluorescein diacetate (DCFH-DA) stock solution (10 μmol/L) was prepared in serum-free cell culture medium. After collecting the cells, they were suspended in the diluted DCFH-DA solution at a concentration of 2×10^7^ cells/mL and incubated at 37 °C for 20 min. The cells were then washed three times with serum-free culture medium to completely remove any unincorporated DCFH-DA. 200 µL of cell suspension containing 1.0×10^6^ cells per well was transferred into a 24-well plate. Then, Peptide-Apt_CD63_, MSC-Exo, or Peptide-Apt_CD63_/Exo (37.4 µM Peptide-Apt_CD63_, 3.8×10^5^ particles/μL MSC-Exo) and Rosup (1 µg/mL) were added to each well, and the plate was incubated at 37 °C for 30 min. Afterwards, the cells were washed three times with PBS for observation using an inverted fluorescence microscope. For flow cytometry analysis, the suspensions of cells after treatment with Peptide-Apt_CD63_/Exo microgels were harvested and analyzed using a BD LSRFortessa flow cytometer.

**1.11.** **In vitro anti-inflammation**

RAW264.7 cells (1.0×10^5^ in 500 μL DMEM containing 10% FBS per well) were seeded in the wells of 24-well plates and cultured, followed by treatment with 100 ng/mL of LPS for 24 h. Microglia (5.0×10^5^ in 500 μL Ham’s F-12 media containing 10% FBS per well) were seeded in the wells of 24-well plates and cultured overnight, followed by treatment with 100 ng/mL of LPS for 24 h. LPS-treated RAW264.7 cells and microglia were then treated with Exo, Peptide-Apt_CD63_, Peptide-Apt_CD63_/Exo (Peptide-Apt_CD63_, 37.4 μM; MSC-Exo, 3.8×10^5^ particles/μL), respectively, for another 24 h. After that, RAW264.7 cells were washed with PBS (pH 7.4) (3×5 min), fixed with 2.5% glutaraldehyde at 37 °C for 15 min, and sealed with immunostaining sealing solution at 37 °C for 60 min. Thereafter, cells were stained with mAb Alexa Fluor^TM^ 488-labeled iNOS (Catalog # 53-5920-82, diluted 1:100, Invitrogen), and mAb APC-labeled Arg-1 (Catalog # 17-3697-82, diluted 1:200, Invitrogen) for 1 h. After washing with PBS (pH 7.4) (3×5 min), nuclei were counterstained with 4’,6-diamidino-2-phenylindole (DAPI). Treated microglia were subjected to sequential immunofluorescence staining. Cells were washed with PBS (pH 7.4) (3×5 min), fixed with 2.5% glutaraldehyde at 37 °C for 15 min, and sealed with immunostaining sealing solution at 37 °C for 60 min. Thereafter, cells were incubated with pAb rabbit anti-Arg-1 (Catalog # GB11285, Servicebio; diluted 1:2000) or pAb rabbit anti-iNOS (Catalog # GB11119, Servicebio; diluted 1:500) overnight at 4 °C. After that, cells were washed with PBS (pH 7.4) (3×5 min), and incubated with an HRP-conjugated goat anti-rabbit IgG (H+L) secondary antibody (Catalog # G1213, Servicebio; diluted 1:2000) at room temperature for 50 min. After thorough washing with PBS (pH 7.4) (3×5 min), tyramide signal amplification (TSA) was performed by applying TSAPLus fluorescent double staining kit (Green iF488+ Red iF594, Servicebio) according to the manufacturer’s instrcutions. After that, nuclei were counterstained with DAPI. Fluorescence images were acquired using an inverted fluorescence microscope (Olympus, Tokyo, Japan). In parallel, the suspensions of cells after treatment with Peptide-Apt_CD63_/Exo were collected and then stained with stained with FITC-conjugated anti-mouse F4/80 (Catalog # 123107, BioLegend; diluted 1:200), PE-conjugated anti-mouse iNOS (Catalog # 696805, BioLegend; diluted 1:160), and PE-conjugated anti-mouse Arg-1 (Catalog # 165803, BioLegend; diluted 1:160) for 30 min at 4 °C in the dark. The expression levels of iNOS and Arg-1 were assessed using a BD LSRFortessa flow cytometer.

**1.12.** **Cell proliferation**

The proliferation of NSCs was determined with Cell Counting Kit-8 (CCK-8) assay. NSCs (8000 cells in 100 μL DMEM/F-12 containing 10% FBS) were seeded into each well of 96-well plate at 37 °C overnight. After that, the cells were incubated with Peptide-Apt_CD63_/Exo microgels at MSC-Exo concentrations varying from 0 to 4.0×10^5^ particles/μL for 24 h. Then, the cell culture medium was discarded and replaced with 100 μL of fresh medium containing 10 μL of CCK-8 for an additional 1 h incubation at 37 ℃. Subsequently, the absorbance at 450 nm was detected using a microplate reader for viability analysis. Each treatment was repeated for 3 times. Then relative cell viability was determined by comparing treated and untreated samples.

**1.13. Cell apoptosis and necrosis**

NSCs (2.0×10^7^ per well) in 6-well plate were treated with MSC-Exo, Peptide-Apt_CD63_, and Peptide-Apt_CD63_/Exo (Peptide-Apt_CD63_, 37.4 μM; MSC-Exo, 3.8×10^5^ particles/μL) for 48 h. Subsequently, the cells were harvested using Accutase enzymatic dissociation to preserve membrane integrity and minimize false-positive apoptosis staining. Apoptotic cells were identified using an Annexin V-FITC/PI Apoptosis Detection Kit (Thermo Fisher Scientific) according to the manufacturer’s instructions. Flow cytometric analysis was conducted on a BD LSRFortessa flow cytometer.

**1.14.** **Cell migration**

The migration of NSCs was evaluated by wound healing assay. NSCs (1.0×10^6^ cells in 1 mL DMEM/F-12 containing 10% FBS) were seeded into PLO-coated 12-well plates and cultivated at 37 °C overnight under the same culture conditions until fully confluent. The attached cells were then incubated with Peptide-Apt_CD63_, MSC-Exo, and Peptide-Apt_CD63_/Exo (Peptide-Apt_CD63_, 37.4 μM; MSC-Exo, 3.8×10^5^ particles/μL), respectively. After 24 h, the cells were washed twice with PBS. Then, the treated cells were cultured in serum-starvation media for 2 h, and a clean wound line on the cell monolayer was generated using a micropipette tip. The cell debris was removed using PBS, and the cells were then free to migrate into the clean area. Wound healing process was observed under an inverted microscope after 24 h.

$$Migration ratio \left( \% \right)=\frac{Cell free area \left( 0 h \right)- Cell free area \left( 24 h \right)}{Cell free area \left( 0 h \right)}\times100\% (1)$$

**1.15.** **Cell differentiation**

NSCs (1.0 × 10^5^ cells in 1 mL DMEM/F12 containing 10% FBS) were seeded into PLO-coated 12-well plates and cultivated at 37 ℃ overnight under the same culture conditions until fully confluent. The attached cells were then incubated with Peptide-Apt_CD63_, MSC-Exo, and Peptide-Apt_CD63_/Exo (Peptide-Apt_CD63_, 37.4 μM; MSC-Exo, 3.8×10^5^ particles/μL), respectively. After 7 days, the cells were collected for RT-qPCR analysis and immunofluorescence staining of Nestin, MAP2, and GFAP expression, as described below.

**1.16.** **Live/dead staining**

HUVECs (1.0×10^5^ cells in 500 μL DMEM containing 10% FBS) were seeded into each well of 24-well plate overnight. After that, PBS, MSC-Exo, Peptide-Apt_CD63_, Peptide-Apt_CD63_/Exo (Peptide-Apt_CD63_, 37.4 μM; MSC-Exo, 3.8×10^5^ particles/μL were used to incubate with cells for 24 h. After incubation, the cells were washed three times with PBS. Before proceeding with cell staining, the staining solution (Calcein AM, 2 µM; PI, 8 µM) was prepared according to the manufacturer’s instructions and sufficient staining solution was added to each well to completely cover the monolayer of cells. The cells were incubated at 37 °C for 20 min. After washing off the staining solution, imaging was conducted using an inverted fluorescence microscope.

**1.17.** **Hemolysis rate**

2 mL of rabbit blood plus 2 mL of normal saline was centrifuged at 2000 rpm for 10 min, and the supernatant was removed after it was clear and slightly yellow. 500 μL of rabbit blood suspended in 10 mL of normal saline. Peptide-Apt_CD63_/Exo solution was diluted in centrifuge tube to prepare 5 concentration gradients ranging from 1 to 16 μg/μL. 1 mL of PBS containing 0.3% Triton was used as a positive control and 1 mL of normal saline was used for negative control. After centrifugation of 1 mL of red blood cell suspension, the supernatant was removed and added to 1 mL of material solution. After incubation at 37 °C for 1 h, the supernatant absorbance was measured and the bottom precipitate was photographed.

$$Hemolysis rate \left( \% \right)=\frac{(A (sample, 540 nm) - A (negative, 540 nm)}{(A (positive, 540 nm) - A(negative, 540 nm)}\times100\% (2)$$

**1.18.** **Surgical procedure and in vivo therapy**

A strike SCI model was established with female C57BL/6 mice (Vital River Laboratory Animal Center, Beijing, China). All the animal experiments were approved by the Institutional Animal Care and Use Committee at Tongji University (approval # TJAF00125102) and carried out according to protocols approved by the committee for animal care and in accordance with the National Ministry of Health. Mice aged 10 weeks were anesthetized with isoflurane (1-2%). The T9-10 dorsal spinal segments were exposed via laminectomy under an operating microscope, and then the T10 level was transected through removal of a 2.0 mm spinal cord segment. The blade was scraped repeatedly along the ventral surface of the spinal canal, and any residual fibers at the lesion site were removed by aspiration, with visual verification to ensure complete transection ventrally and laterally. The T9 and T11 were fixed on the Impactor model-III spinal cord percussion platform by fixed forceps. The application program of the Impactor model-III spinal cord percussion device was set to 9 cm. The percussion rod was adjusted to the preset height and close the safety. The strike rod was released so that it falls vertically along the casing, the stopwatch was released at the same time, check whether the strike position is in the middle of the spinal cord, and then the firing pin was lifted when it remains stationary for 3 s after impact. The criteria for success were that the injury site was located in the middle of the spinal cord, along with spinal cord congestion and edema, and tail wagging reflex. After awakening from anesthesia, both legs of mouse became paralyzed. Suture the muscle layer, the fascia layer and the skin layer successively. After the suture, disinfect the operative part with alcohol and wipe away the blood to prevent each other from biting the thread. Six days later, the spinal cord site was re-exposed according to the above steps, and the spinal cord injury site was injected using a stereotaxic device connected to a syringe. The SCI mice were divided into four groups (n = 6), and each was injected with 2 μL of PBS, Peptide-Apt_CD63_, MSC-Exo, and Peptide-Apt_CD63_/Exo (Peptide-Apt_CD63_, 374 μM; MSC-Exo, 3.8×10^6^ particles/μL). The mice underwent laminectomy without spinal cord percussion was used as sham control. After the operation, each mouse was massaged into its bladder once a day for urination. The mice were kept at a temperature of 24-26 °C and relative humidity of 35-45% on a 12 h light/dark cycle. The in vivo safety assessment of the materials was conducted through H&E staining of heart, liver, spleen, lung, and kidney tissues in the mouse model after treatment with Peptide-Apt_CD63_/Exo microgels for 7 days, with comparison to normal control group.

**1.19.** **Behavioral assessment**

The Basso Mouse Scale (BMS) open-field ratings were assessed weekly by two independent observers blinded to the group identity. The extent of motor function recovery after SCI is quantified by observing indicators such as hindlimb joint movements, trunk stability, and gait coordination in mice. The gait scores ranging from 0 (paralysis) to 9 (normal) are listed in Table S1. Recovery of hindlimb movements was assessed for a duration of 6 weeks. Left and right hindlimb movements were scored separately using the BMS and averaged. Each experimental group undergoes triplicate assessments with three individual mice.

**1.20.** **Gait analysis**

The small animal gait analysis system is activated with the treadmill speed (Sansbio SA101C, Nanjing, China) sequentially set to 5, 10, and 15 m/min for individual trials lasting 3-5 min. A stimulation intensity ranging from 0.8 to 1.5 mA is maintained throughout the procedure. The mouse is positioned on the treadmill before system initiation, and the trial is terminated when [≥](https://baike.baidu.com/item/%E2%89%A5/8288688) 5 consecutive locomotor steps are captured, followed by immediate data acquisition. Multiple kinematic parameters are quantified for each complete step cycle through automated analysis. Each experimental group undergoes triplicate testing sessions with three individual mice.

**1.21.** **Electrophysiological studies**

Electrophysiological assays were performed 6 weeks after the operation. A 2% chloral hydrate solution was administered intraperitoneally to the mice at a dose of 2 mL/100 g. Mice were secured in a stereotaxic apparatus, followed by shaving of the head fur, removal of the scalp, and clearance of connective tissue to expose the bregma and surrounding cranial bones. A craniotomy was performed 2-3 mm anterior and posterior to the bregma, and 3-4 mm lateral to the midline (targeting the sensorimotor cortex). The hemisphere selection (left/right) for craniotomy correlated with the recorded hindlimb side: left-hemisphere craniotomy was performed when recording signals from the right hindlimb. The AlphaLab SnR^TM^ ground electrode was inserted into the abdominal cavity, the recording electrode into the hindlimb, and the stimulating electrode was placed on the dura mater, with the stereotaxic frame serving as the electrical ground. Signals were recorded and stored. MEP can comprehensively reflect the locomotor function of an animal and indicate neurological recovery in SCI.

**1.22.** **Histopathological staining**

Histopathological staining includes H&E staining, LFB staining, and Nissl staining. Mice were anesthetized, and a heart perfusion with PBS and 4% paraformaldehyde was performed. Subsequently, a total of 1 cm of the spinal cord segment (T10 level) was excised and embedded in paraffin. Sections were prepared at a thickness of 3 µm using a microtome (Leica RM2016, Germany).

For Hematoxylin and Eosin (H&E) staining, sections were deparaffinized through the following sequence: Eco-friendly deparaffinization fluid I for 20 min, followed by fluid II for 20 min, anhydrous ethanol I for 5 min, anhydrous ethanol II for 5 min, and 75% ethanol for 5 min. Afterwards, sections were washed with distilled water and treated with high-definition constant-dye pre-treatment solution for 1 min. H&E staining was performed by immersing the sections in hematoxylin for 3-5 min, followed by washing with distilled water, differentiation, and subsequent bluing. Sections were dehydrated in 95% ethanol for 1 min and stained with eosin for 15 s. Finally, the sections underwent dehydration and mounting in the following sequence: anhydrous ethanol I for 2 min, anhydrous ethanol II for 2 min, anhydrous ethanol III for 2 min, n-butanol I for 2 min, n-butanol II for 2 min, xylene I for 2 min, and xylene II for 2 min, followed by mounting with neutral gum. All reagents were obtained from Servibio.

For LFB staining, sections were deparaffinized as described above. Luxol Fast Blue Solution A (Servicebio) was preheated in an oven at 60 °C for 30 min. Sections were incubated in Luxol Fast Blue Solution A for 1 hour, followed by a rapid rinse with distilled water. The sections were then differentiated in warm Luxol Fast Blue Solution B for 2 s and immediately differentiated in solution C for 15 s. Differentiation was monitored under a microscope until the myelin appeared with a blue background and near translucency. Sections were then dried in a 65 °C oven (approximately 30 min) until the slides were dry and subsequently stained with eosin after being rehydrated in 95% ethanol. The slides were finally dehydrated and mounted using xylene as described for the H&E staining. Dehydration sealing reagents were purchased from Sinopharm Chemical Reagent Co. Ltd.

For Nissl staining, sections were deparaffinized following the H&E protocol and immersed in Nissl staining solution (Servicebio) for 2-5 min. They were then washed, slightly differentiated in 0.1% acetic acid, and rinsed with distilled water to halt the reaction. The degree of differentiation was confirmed under a microscope before the sections were dried in an oven. Finally, sections were cleared in clean xylene for 10 min and mounted with neutral gum. All stained sections were imaged and analyzed using an inverted fluorescence microscope.

**1.23. Immunofluorescence staining**

Following the induction of anesthesia in mice, a heart perfusion with PBS and 4% paraformaldehyde was performed. After dissection, a total of 1 cm of spinal cord tissue encompassing the T10 segment was excised and embedded in paraffin. The sections were then subjected to deparaffinization in the following sequence: Eco-friendly deparaffinization solution I for 10 min, solution II for 10 min, and solution III for 10 min, followed by immersion in anhydrous ethanol I for 5 min, anhydrous ethanol II for 5 min, and anhydrous ethanol III for 5 min. The sections were washed with distilled water. Following the induction of anesthesia in mice, a heart perfusion with PBS and 4% paraformaldehyde was performed. After dissection, a total of 1 cm of spinal cord tissue encompassing the T10 segment was excised and embedded in paraffin. The sections were then subjected to deparaffinization in the following sequence: Eco-friendly deparaffinization solution I for 10 min, solution II for 10 min, and solution III for 10 min, followed by immersion in anhydrous ethanol I for 5 min, anhydrous ethanol II for 5 min, and anhydrous ethanol III for 5 min. The sections were washed with distilled water. The slides were washed three times with PBS (pH 7.4) on a shaking platform, with each wash lasting 5 min. After briefly shaking off excess PBS, a hydrophobic pen was used to outline the tissue area on each slide. Bovine serum albumin (BSA) was then applied for blocking: 10% donkey serum for the primary antibody sourced from goat and 3% BSA for the other primary antibodies, allowing for blocking for 30 min. The appropriate primary antibodies from different sources were mixed and then added to the sections, which were incubated overnight at 4 °C in a humidified chamber. Following this incubation, the slides were washed again three times with PBS (pH 7.4) for 5 min each. The corresponding secondary antibodies were applied and incubated for 50 min at room temperature, protected from light. After another set of washes in PBS, DAPI staining solution was added, and the sections were incubated at room temperature for 10 min in the dark. The slides were washed three more times with PBS, and a fluorescent quenching solution was applied for 5 min before washing with running water for 10 min. Finally, the slides were mounted with an antifade mounting medium. Fluorescent images were obtained using inverted fluorescence microscope. The used primary antibodies against iNOS, Arg-1, Iba-1, Nestin, MAP2, and GFAP are pAb rabbit anti-iNOS (Catalog # GB11119, diluted 1:500, Servicebio), pAb rabbit anti-Arg-1 (Catalog # GB11285, diluted 1:2000, Servicebio), mAb mouse anti-Iba-1 (Catalog # GB15105, diluted 1:500), mAb mouse anti-Nestin (Catalog # GB13432, diluted 1:1000, Servicebio), pAb rabbit anti-MAP2 (Catalog # GB115559, diluted 1:500, Servicebio), mAb mouse anti-GFAP (Catalog # GB15096, diluted 1:500, Servicebio), respectively. The used secondary antibody for Arg-1 and iNOS is Cy3-labeled goat anti-rabbit IgG (H+L) (Catalog # GB21303, diluted 1:500, Servicebio). The used secondary antibody for Iba-1 is Alexa Fluor 488-labeled goat anti-mouse IgG (H+L) (Catalog # GB25301, diluted 1:400, Servicebio). The used secondary antibody for Nestin and GFAP is Cy3-labeled goat anti-mouse IgG (H+L) (Catalog # GB21301, diluted 1:300, Servicebio). The used secondary antibody for MAP2 is Alexa Fluor 488-labeled goat anti-rabbit IgG (H+L) (Catalog # GB25303, diluted 1:400, Servicebio). Nuclei were stained with 4’,6-diamidino-2-phenylindole (DAPI).

**1.24. RT-qPCR analysis**

Total RNA was then extracted using the RNAeasy^TM^ Animal RNA Isolation Kit with Spin Column (R0026, Beyotime). For the spinal cord tissue, magnetic beads and 1 mL of lysis buffer in the kit were added into a centrifuge tube. Then, the tube was grinded for 30 seconds using a fully automatic sample cryogenic grinder (Jingxin, JXFSTPRP-CL, Shanghai, China). For the cells, 300 μL of lysis buffer in the kit were added to a centrifuge tube. The lysates were gently pipetted up and down 8-10 times until the pellet was dissolved and the solution became clear. The lysates were transferred to a clean centrifuge tube for further purification and collection according to the manufacturer’s instructions. First-strand cDNA was synthesized using the BeyoRT^TM^ III First Strand cDNA Synthesis Kit (D7178S, Beyotime) according to the manufacturer’s instructions. The prepared cDNA was mixed with specific primers (Table S2) and BeyoFast^TM^ SYBR Green qPCR Mix (2×, Low ROX) to detect the expression levels of TNF-α, MMP9, IL-10, TGF-β, Nestin, MAP2, and GFAP genes by ABI7500 PCR system (Applied Biosystems, Waltham, MA). The amplification was performed for 40 cycles with fluorescence detection using SYBR Green. Each cycle consisted of denaturation at 95 °C for 15 s, annealing at 62 °C for 15 s, and extension at 72 °C for 30 s. Relative gene expression was calculated using the comparative cycle threshold (ΔΔCt) method with GAPDH as the reference gene. For each sample, the measurement was repeated for 3 times.

**Table S1.** BMS scoring scale (0-9).

| Score | Scoring criteria |
| --- | --- |
| 0 | No ankle movement |
| 1 | Slight ankle movement. Slight: Moves less than half of the ankle joint excursion. |
| 2 | Extensive ankle movement. Extensive: Moves more than half of the ankle joint excursion. |
| 3 | Plantar placing of the paw with or without weight support - OR Occasional, frequent or consistent dorsal stepping but no plantar stepping. Plantar placing: Paw is actively placed with both the thumb and the last toe of the paw touching the ground. Weight support: (dorsal or plantar): The hindquarters must be elevated enough that the hind end near the base of the tail is raised off the surface and the knees do not touch the ground during the step cycle. |
| 4 | Occasional plantar stepping. Occasional: Stepping less than or equal to half of the time moving forward. |
| 5 | Frequent or consistent plantar stepping, no coordination - OR Frequent or consistent plantar stepping, some coordination, paws rotated at initial contact and lift off (R/R). Frequent: Stepping more than half the time moving forward. |
| 6 | Frequent or consistent plantar stepping, some coordination, paws parallel at initial contact (P/R, P/P) - OR Frequent or consistent plantar stepping, mostly coordinated, paws rotated at initial contact and lift off (R/R). |
| 7 | Frequent or consistent plantar stepping, mostly coordinated, paws parallel at initial contact and rotated at lift off (P/R) - OR Frequent or consistent plantar stepping, mostly coordinated, paws parallel at initial contact and lift off (P/P), and severe trunk instability. |
| 8 | Frequent or consistent plantar stepping, mostly coordinated, paws parallel at initial contact and lift off (P/P), and mild trunk instability - OR Frequent or consistent plantar stepping, mostly coordinated, paws parallel at initial contact and lift off (P/P), and normal trunk stability and tail down or up & down. |
| 9 | Frequent or consistent plantar stepping, mostly coordinated, paws parallel at initial contact and lift off (P/P), and normal trunk stability and tail always up. |

**Table S2.** Lists of RT-qPCR primers.

| Gene | Forward Strand (5’-3’) | Reverse Strand (5’-3’) |
| --- | --- | --- |
| Nestin | AGAGAAGACAGTGAGGCAGATGAG | GAGGCAGGAGACTTCAGGTAGAG |
| GAPDH | AGTCCACTGGCGTCTTCACC | TGATCTTGAGGCTGTTGTCATACTTC |
| TNF-α | TCAGCAAGGACAGCAGAGGAC | GGTGGAGCCGTGGGTCAG |
| MAP2 | GGCGAAGGATAAAGTCACTGATGG | GGCGAAGGATAAAGTCACTGATGG |
| iNOS | CAACTACTGCTGGTGGTGACAAG | CTGAAGGTGTGGTTGAGTTCTCTAAG |
| Arg-1 | AAGACAGCAGAGGAGGTGAAGAG | TAGTCAGTCCCTGGCTTATGGTTAC |
| GFAP | GAACAACCTGGCTGCGTATAGAC | TCTCCTCCTCCAGCGATTCAAC |
| MMP9 | AGTATCTGTATGGTCGTGGCTCTAAG | GGAGGTGCTGTCGGCTGTG |
| TGF-β | CAACAATTCCTGGCGTTACCTTGG | TGTATTCCGTCTCCTTGGTTCAGC |
| IL-10 | GGTTGCCAAGCCTTATCGGAAATG | GCCGCATCCTGAGGGTCTTC |

2. Supplementary Figures


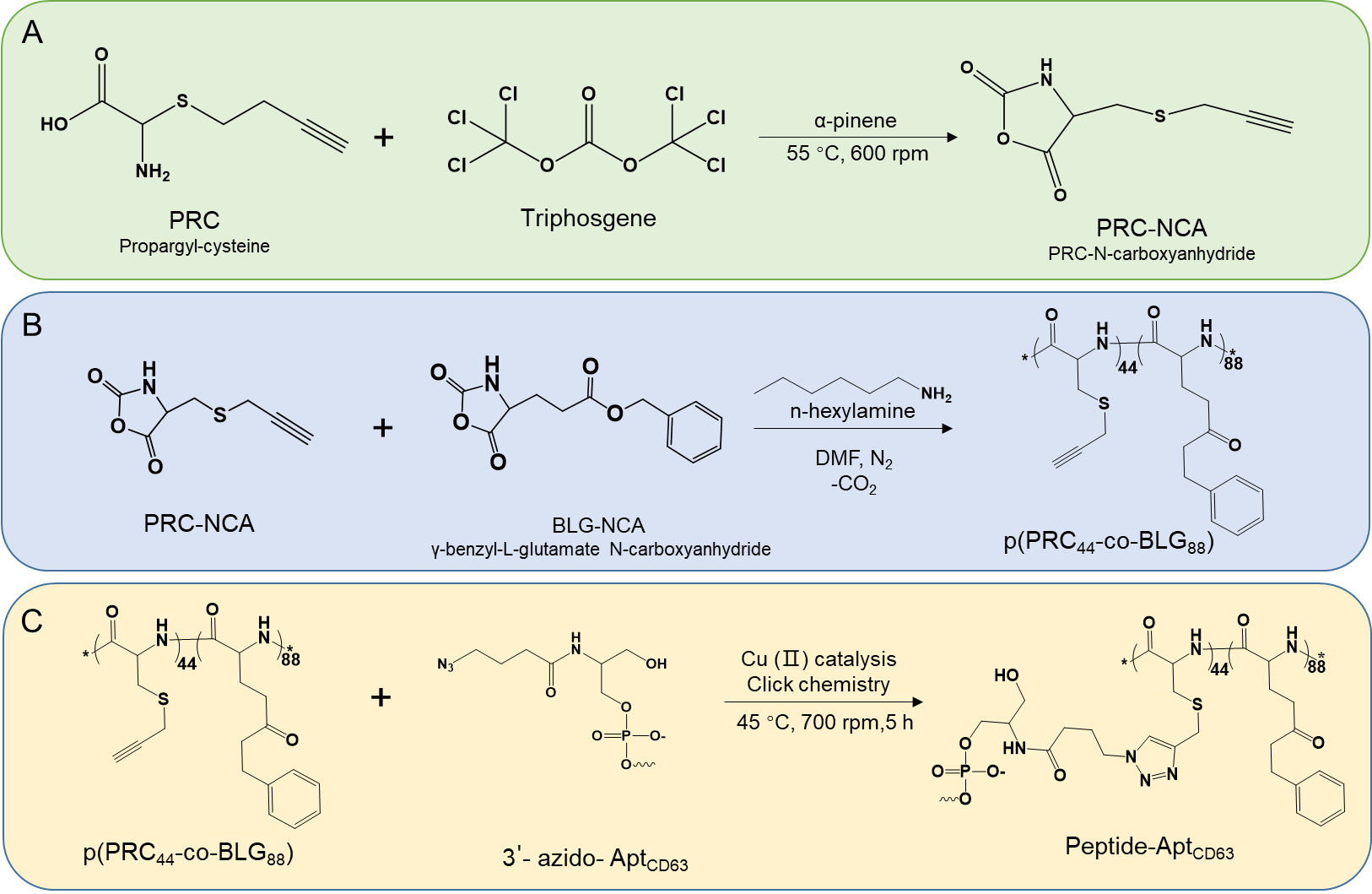


**Figure S1.** Scheme illustration of synthesis of (A) PRC-NCA, (B) polypeptide via ROP of NCA, and (C) Peptide-Apt_CD63_ conjugate via click chemistry. Following the established methods, the yield of PRC-NCA was estimated to be approximately 75%.


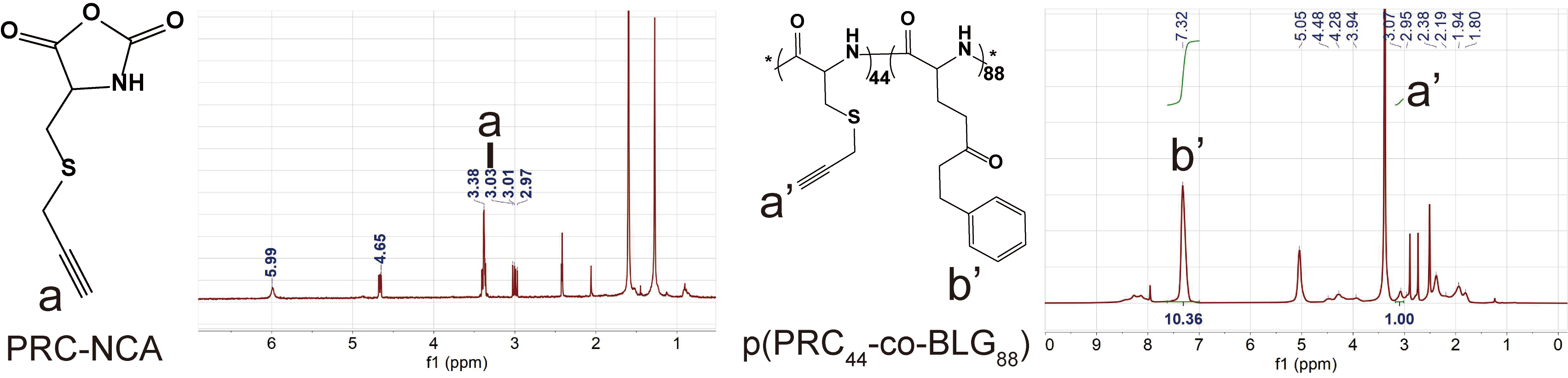


**Figure S2*.*** ^1^H NMR spectra of PRC-NCA and polypeptide. The ratio of phenyl ring to alkyne group was calculated to be approximately 2 : 1.


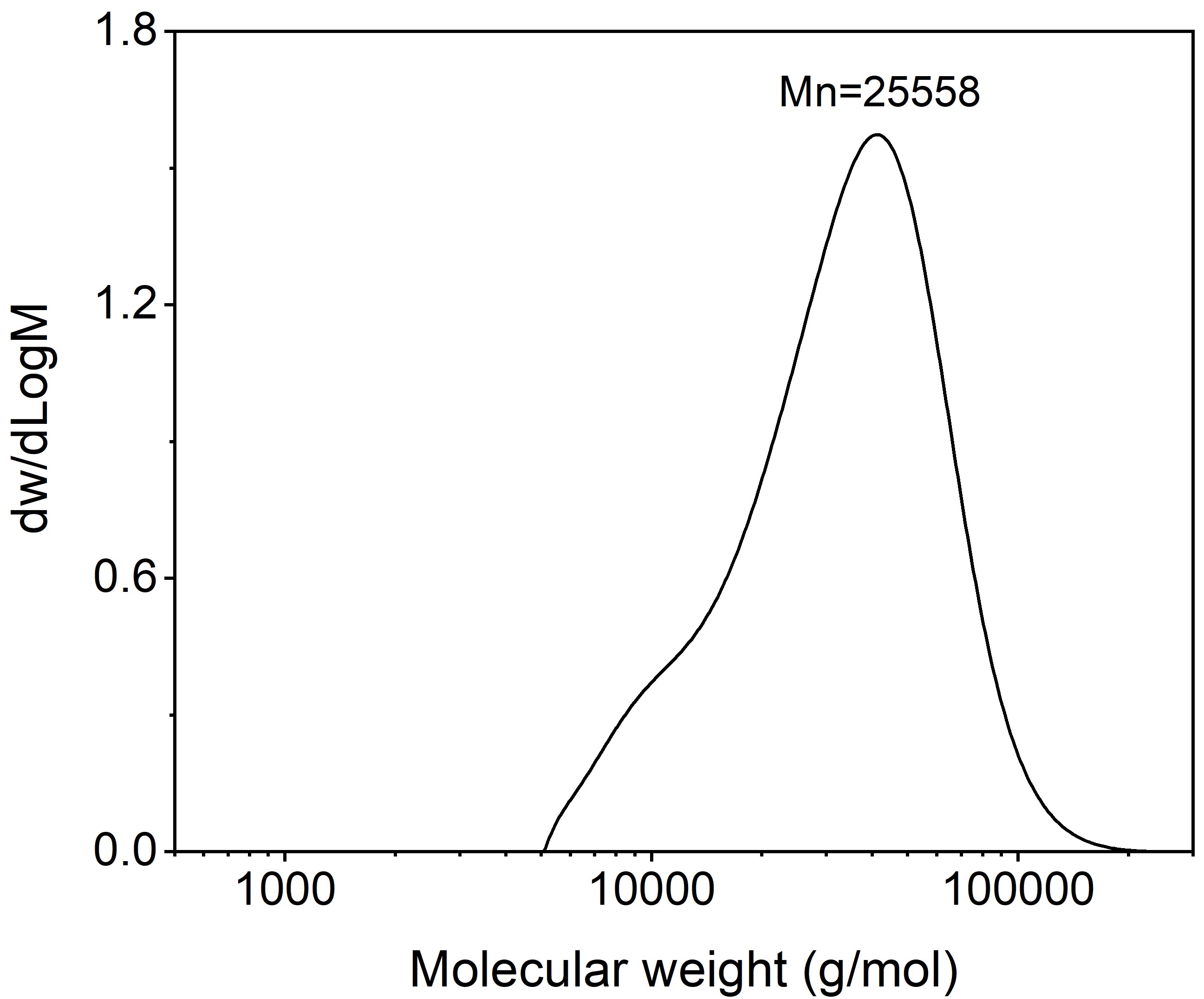


**Figure S3.** GPC analysis of molecular weight distribution of polypeptide, revealing M_n_ = 25558 Da and PDI = 1.5.


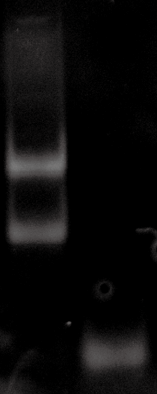


**Figure S4.** 6% of PAGE analyzing Peptide-Apt_CD63_ conjugate (left lane) and azido-Apt_CD63_ (right lane). Note that, the average number of Apt_CD63_ moieties grafted onto each polypeptide chain was between 2.0-3.0, as determined by measuring the unbonded Apt_CD63_ with a microspectrophotometer after ultrafiltration. Therefore, the upper band in left lane represents peptides grafted with three aptamers, while the lower band corresponds to those with two aptamers.

**Figure S5.** Zeta potentials of MSC-Exo and Milk-Exo.


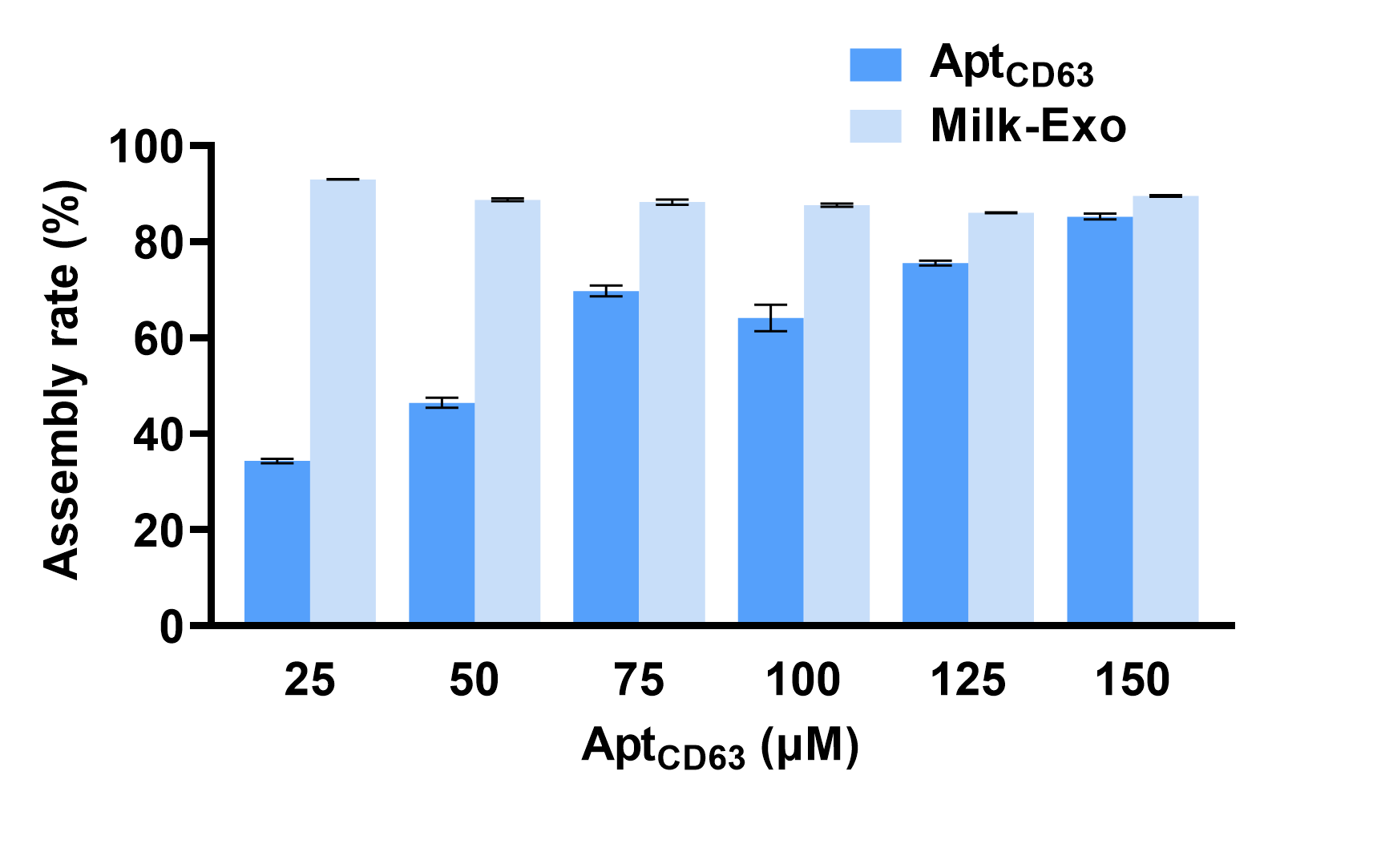


**Figure S6.** Assembly rate of peptide-tethered Apt_CD63_ and Milk-Exo with different concentrations of peptide-tethered Apt_CD63_ (25-150 μM) and a constant concentration of Milk-Exo (9.0×10^8^ particles/μL) after 20 h of co-incubation.


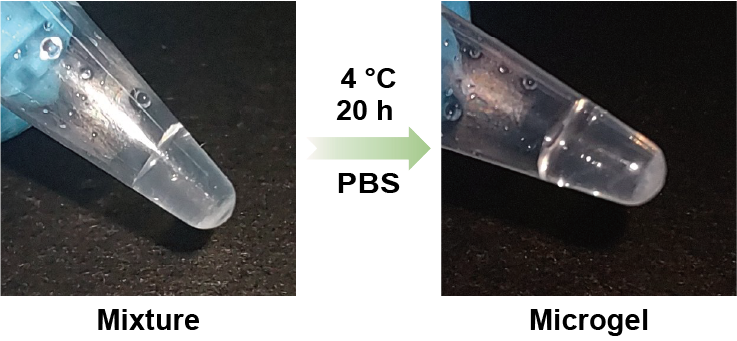


**Figure S7.** Photographs of the mixture of Peptide-Apt_CD63_ (125 μM Apt_CD63_) and Milk-Exo (9.0×10^8^ particles/μL) before and after assembly.


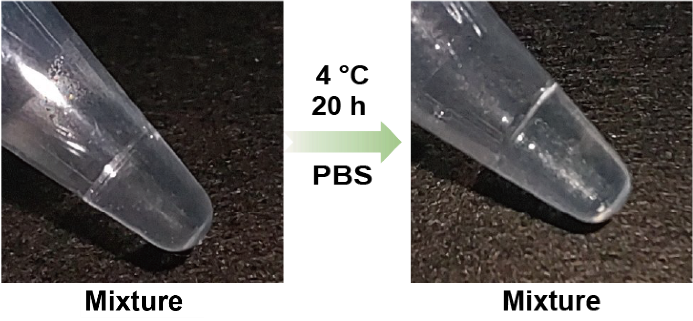


**Figure S8.** Photographs of the mixture of Peptide-Apt_CD63_ (125 μM Apt_CD63_) and Milk-Exo (5.0×10^5^ particles/μL) before and after incubation.

**Figure S9.** The expression levles of CD63 on MSC-Exo and Milk-Exo.


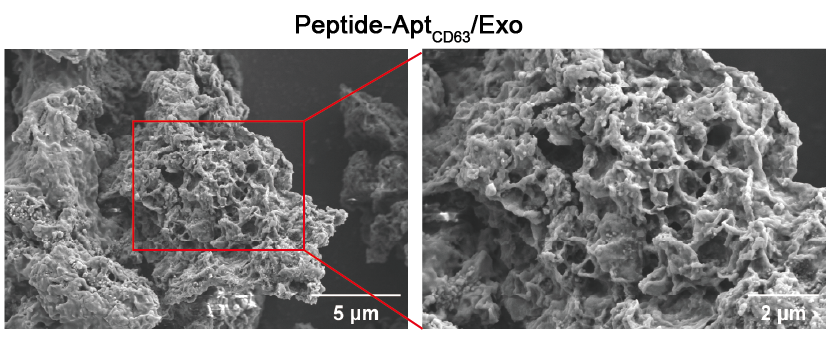


**Figure S10.** SEM images of Peptide-Apt_CD63_/Exo microgels. The microgels exhibited a 3D cross-linked network structure.


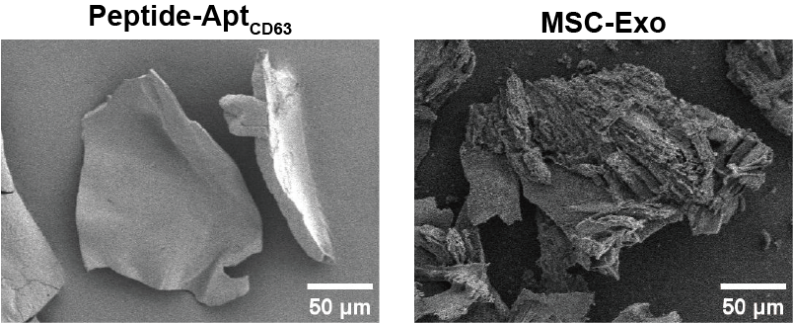


**Figure S11.** SEM images of Peptide-Apt_CD63_ and MSC-Exo. Note that, Peptide-Apt_CD63_ conjugate exhibited a membrane-like structure, whereas MSC-Exo formed large dense aggregates. In contrast, their assembly exhibited a three-dimensional porous structure (Figures 1F, S10).

**Figure S12.** Size distribution of Peptide-Apt_CD63_/Exo microgels.


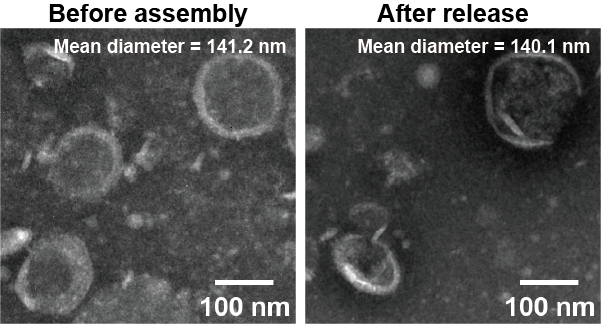


**Figure S13.** TEM images of MSC-Exo before assembly and after release from microgels.

**Figure S14.** The expression levels of CD63 on MSC-Exo before assembly and after release from microgels. The expression level of CD63 on MSC-Exo after release was 94.5% of that before assembly.

**Figure S15.** Cumulative release profiles of MSC-Exo after incubation of Peptide-Apt_CD63_/Exo microgels with normal saline and FBS up to 12 h, respectively.


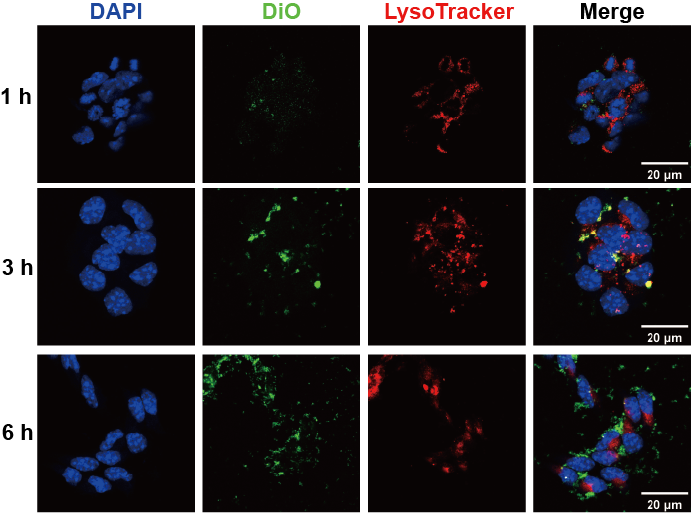


**Figure S16.** Time-lapse confocal microscopy imaging of LysoTracker Red-stained NSCs after treatment with DiO-labeled Peptide-Apt_CD63_/Exo microgels (Peptide-Apt_CD63_, 37.4 μM; MSC-Exo, 3.8×10^5^ particles/μL) for 6 h.


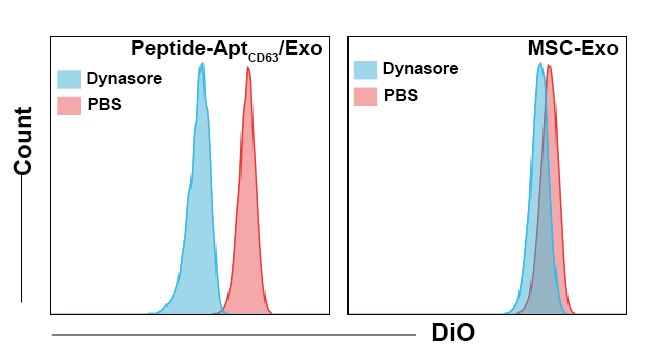


**Figure S17.** Flow cytometry analysis of endocytosis of Peptide-Apt_CD63_/Exo microgels and MSC-Exo by NSCs pre-treated with dynasore (Peptide-Apt_CD63_, 37.4 μM; MSC-Exo, 3.8×10^5^ particles/μL).


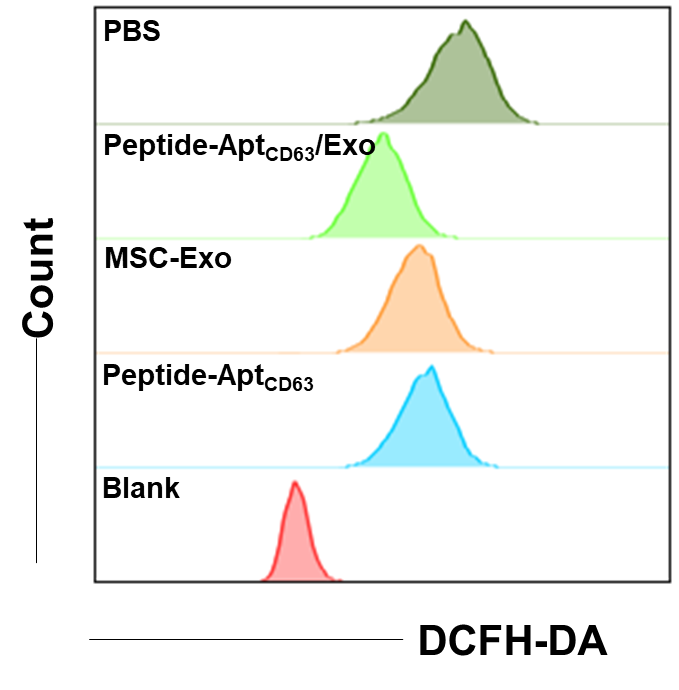


**Figure S18.** Flow cytometry analysis of intracellular ROS using an ROS indicator, DCFH-DA, in NSCs after different treatments (Peptide-Apt_CD63_, 37.4 μM; MSC-Exo, 3.8×10^5^ particles/μL). In comparison to the PBS group, both MSC-Exo and Peptide-Apt_CD63_ treatments reduced DCFH-DA fluorescence signals. The Peptide-Apt_CD63_/Exo group displayed the weakest signal, indicating the most significant intracellular ROS scavenging capability.


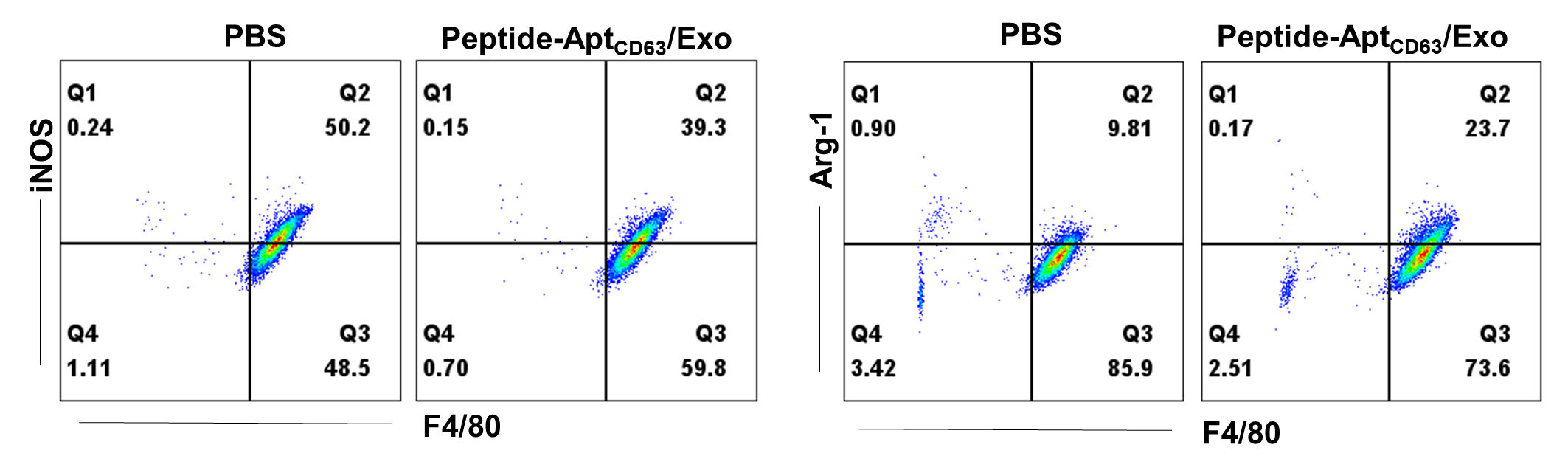


**Figure S19.** Flow cytometry analysis of LPS-activated microglia after treatment with Peptide-Apt_CD63_/Exo microgels (Peptide-Apt_CD63_, 37.4 μM; MSC-Exo, 3.8×10^5^ particles/μL) for 24 h. The proportion of M1 phenotype was decreased and the proportion of M2 phenotype was elevated by Peptide-Apt_CD63_/Exo compared to PBS.


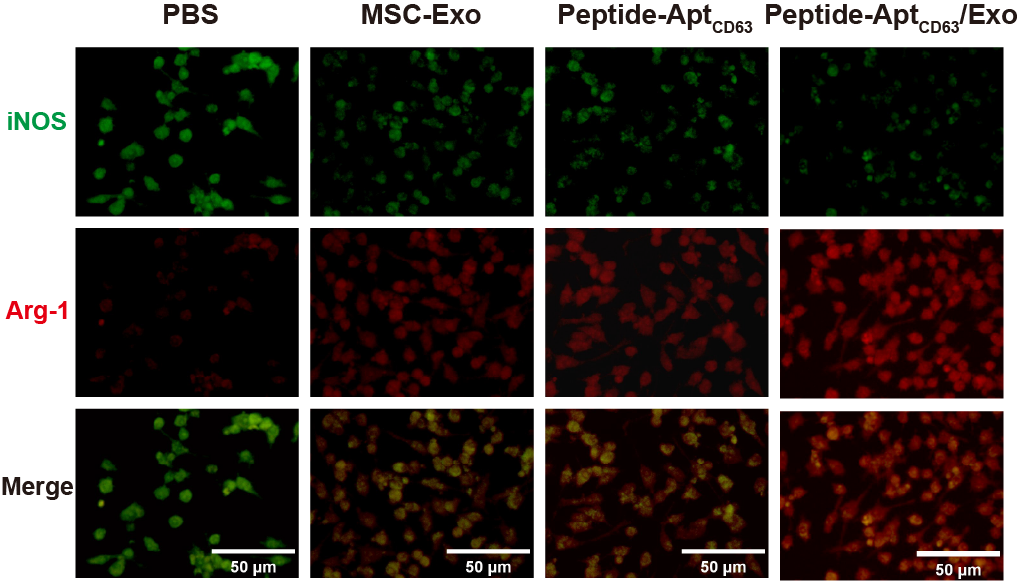


**Figure S20.** Immunofluorescence staining of iNOS and Arg-1 in LPS-activated macrophages (RAW 264.7 cells) after different treatments (Peptide-Apt_CD63_, 37.4 μM; MSC-Exo, 3.8×10^5^ particles/μL) for 24 h. The green fluorescence intensity of immunostained iNOS was weakened and the red fluorescence intensity of immunostained Arg-1 was elevated by MSC-Exo and Peptide-Apt_CD63_ compared to PBS alone. Nevertheless, the Peptide-Apt_CD63_/Exo group displayed the most weakened green fluorescence and the most elevated red fluorescence.


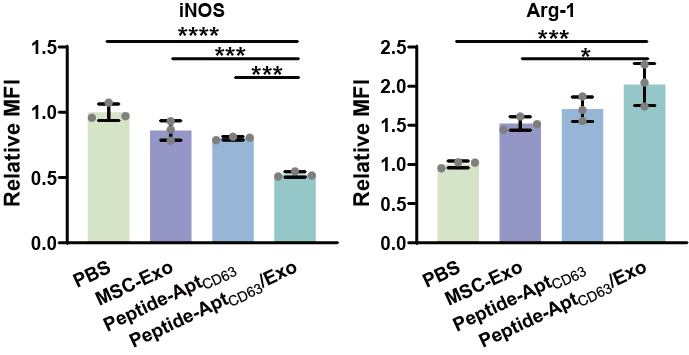


**Figure S21.** Relative mean fluorescence intensity (MFI) of immunostained iNOS and Arg-1 in LPS-activated macrophages after different treatments for 24 h in Figure S20.


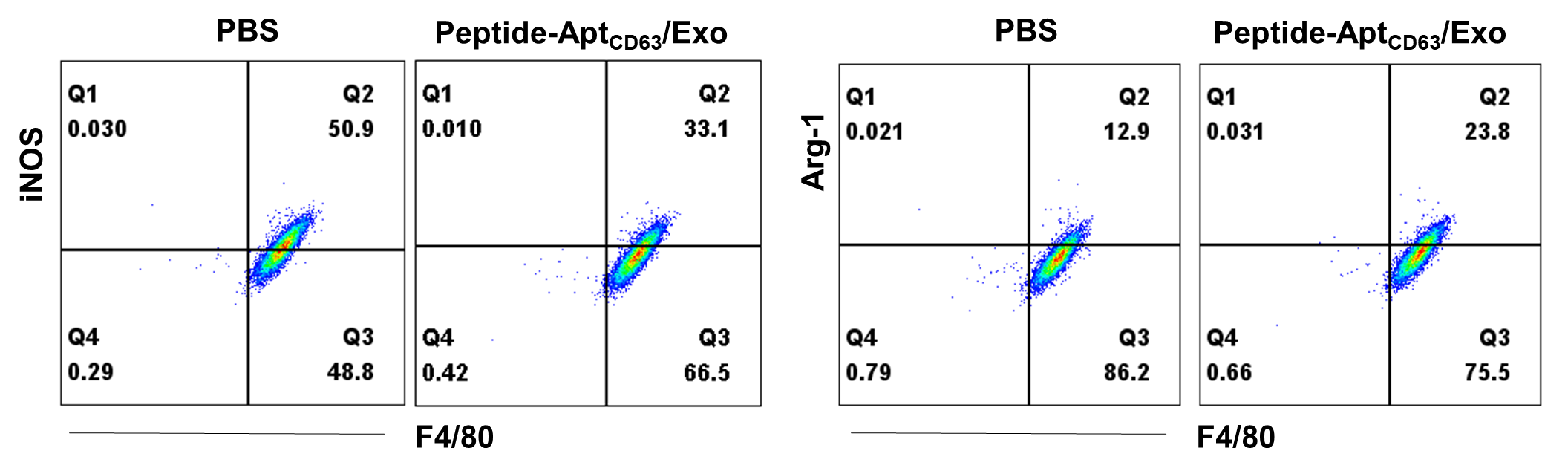


**Figure S22.** Flow cytometry analysis of LPS-activated macrophages after treatment with Peptide-Apt_CD63_/Exo microgels (Peptide-Apt_CD63_, 37.4 μM; MSC-Exo, 3.8×10^5^ particles/μL) for 24 h. The proportion of M1 phenotype was decreased and the proportion of M2 phenotype was elevated by Peptide-Apt_CD63_/Exo compared to PBS.


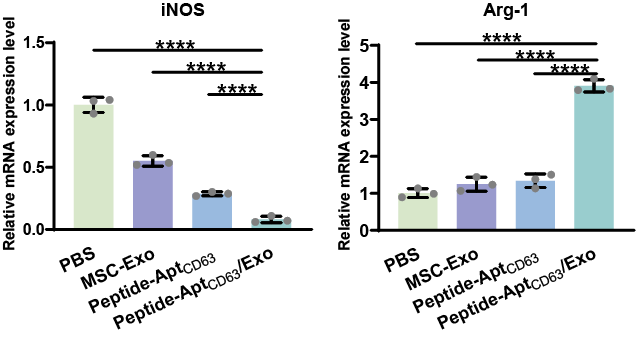


**Figure S23.** The mRNA expression level of iNOS and Arg-1 in LPS-activated macrophages. The green fluorescence intensity of immunostained iNOS was weakened and the red fluorescence intensity of immunostained Arg-1 was elevated by MSC-Exo and Peptide-Apt_CD63_ compared to PBS alone. Nevertheless, the Peptide-Apt_CD63_/Exo group displayed the most weakened green fluorescence and the most elevated red fluorescence. The iNOS mRNA expression level was downregulated by MSC-Exo (~0.55-fold), Peptide-Apt_CD63_ (~0.29-fold), and Peptide-Apt_CD63_/Exo (~0.08-fold), whereas the mRNA expression level of Arg-1 was upregulated by MSC-Exo (~1.25-fold), Peptide-Apt_CD63_ (~1.34-fold), and Peptide-Apt_CD63_/Exo (~3.91-fold) respectively, as compared to the PBS group.


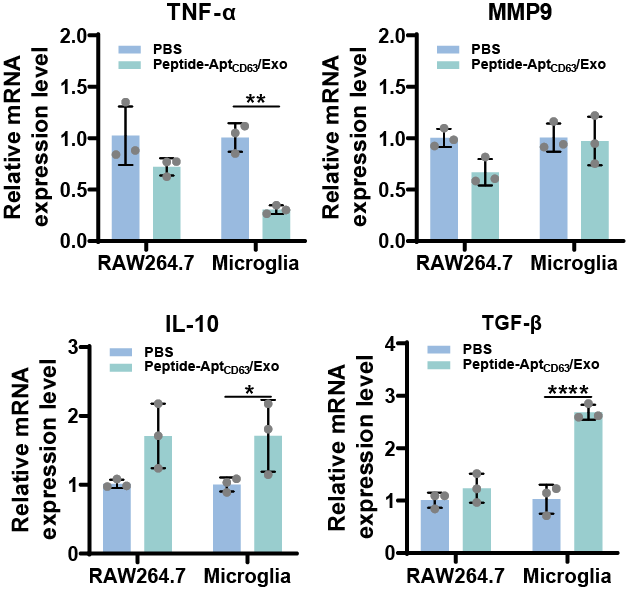


**Figure S24.** Relative mRNA expression levels of TNF-α, MMP9, IL-10, and TGF-β in LPS-activated macrophages and microglia after treatment with Peptide-Apt_CD63_/Exo (Peptide-Apt_CD63_, 37.4 μM; MSC-Exo, 3.8×10^5^ particles/μL) for 24 h. Note that, the results indicated that Peptide-Apt_CD63_/Exo microgels markedly downregulated the expression levels of pro-inflammatory TNF-α (macrophages, ~0.72-fold; microglia, ~0.31-fold) and MMP9 (macrophages, ~0.67-fold; microglia, ~0.97-fold), and simultaneously upregulated anti-inflammatory factors like IL-10 (macrophages, ~1.71-fold; microglia, ~1.70-fold) and TGF-β (macrophages, ~1.23-fold; microglia, ~2.69-fold) as compared to the PBS group.


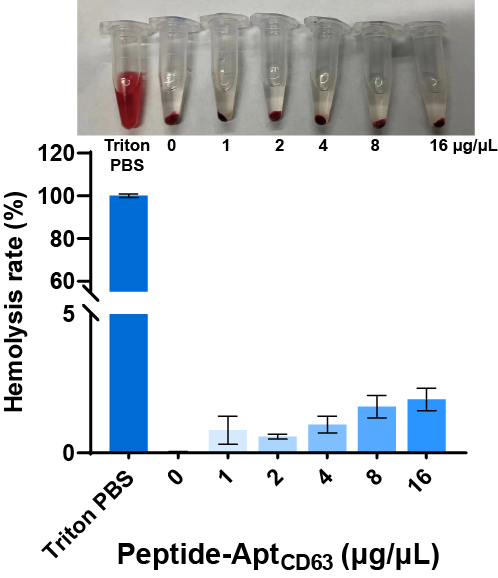


**Figure S25.** The hemolysis rate of Peptide-Apt_CD63_/Exo microgels at different concentrations of Peptide-Apt_CD63_. Note that, the hemolysis rate of Peptide-Apt_CD63_/Exo microgels was below 5%, demonstrating their good blood compatibility at the studied concentration range.


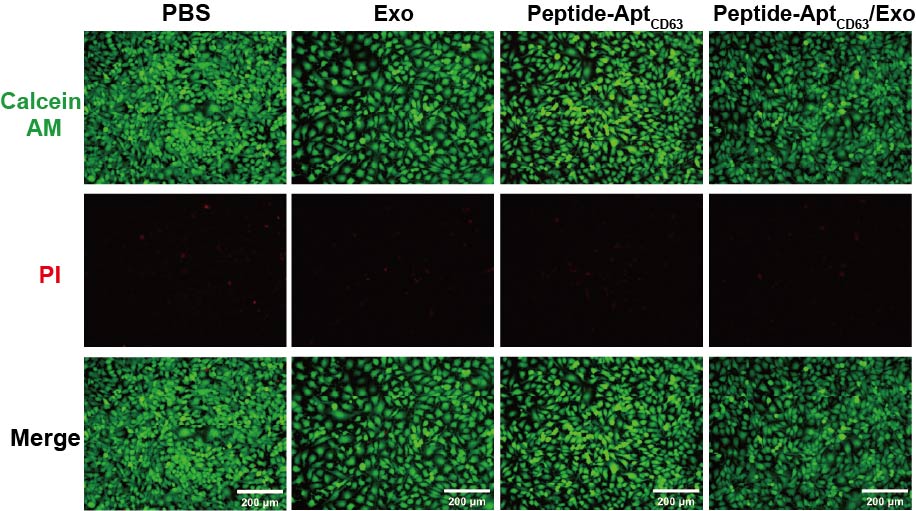


**Figure S26.** Live/dead staining of HUVECs after treated with different materials (Peptide-Apt_CD63_, 37.4 μM; MSC-Exo, 3.8×10^5^ particles/μL) for 24 h. The viable cells were stained in green by Calcein AM, while the dead cells were stained in red by propidium iodide (PI). Note that, negligible dead cells were observed following culture with MSC-Exo, Peptide-Apt_CD63_, and Peptide-Apt_CD63_/Exo microgels, highlighting their excellent cytocompatibility.


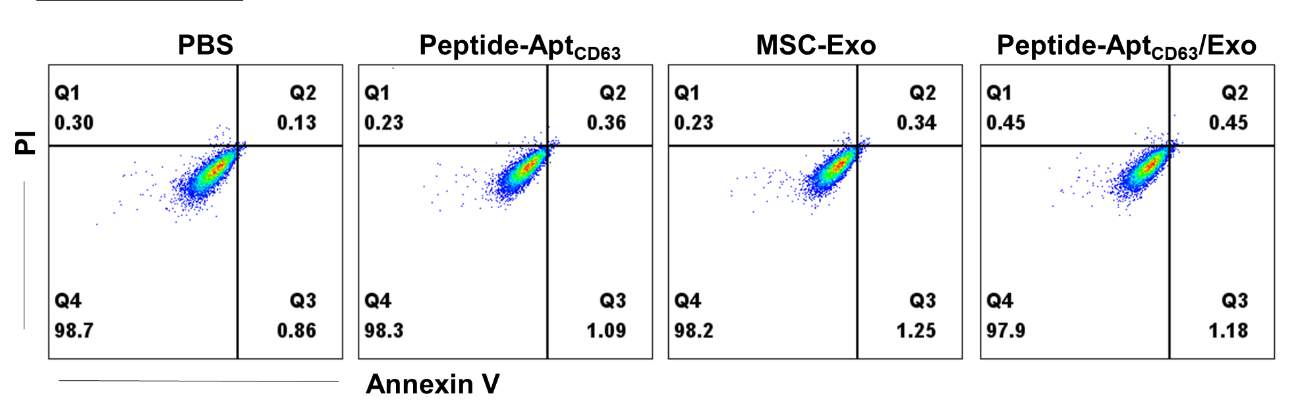


**Figure S27.** Flow cytometry analysis of apoptosis and necrosis after different treatments (Peptide-Apt_CD63_, 37.4 μM; MSC-Exo, 3.8×10^5^ particles/μL) for 24 h. Note that, the proportions of Annexin V+, PI+, and Annexin V/PI+ cells in the Peptide-Apt_CD63_/Exo group were as low as those of the PBS group, suggesting negligible apoptosis or necrosis.


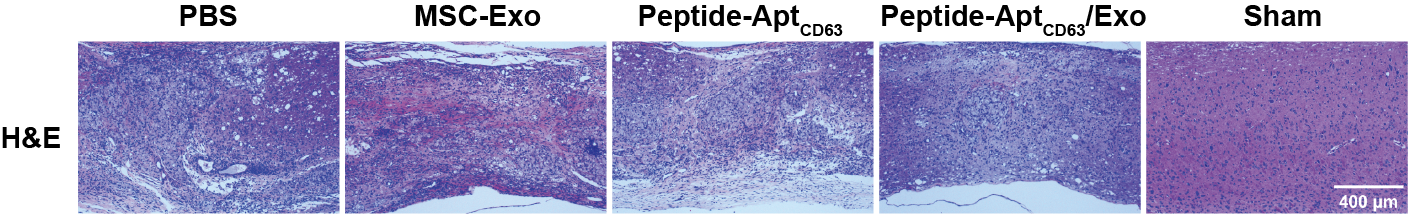


**Figure S28.** H&E staining of the injury sites in different groups at 4 weeks post-surgery. Note that, the PBS group exhibited notable swelling, severe rupture, and extensive vacuolation at the lesion site. While neither the Exo nor Peptide-Apt_CD63_ groups presented with swollen structures, both still exhibited severe rupture and numerous vacuolar structures and even displayed spinal cord atrophy. However, those pathological features were significantly alleviated in the Peptide-Apt_CD63_/Exo group. The peptide-Apt_CD63_/Exo group displayed minimal swelling or atrophy, and dense structural integrity with few small vacuoles. These findings suggest a superior therapeutic efficacy of microgels at 4 weeks post-surgery.


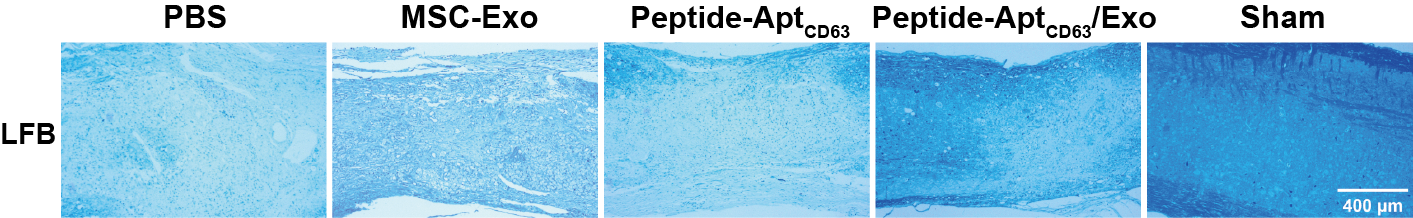


**Figure S29.** LFB staining of the injury sites in different groups at 4 weeks post-surgery. Note that, following SCI, significant demyelination was evident across all SCI groups, as indicated by LFB staining images. While the Exo group exhibited slight myelin regeneration, the Peptide-Apt_CD63_/Exo group displayed a marked increase in myelin content within the injury region.


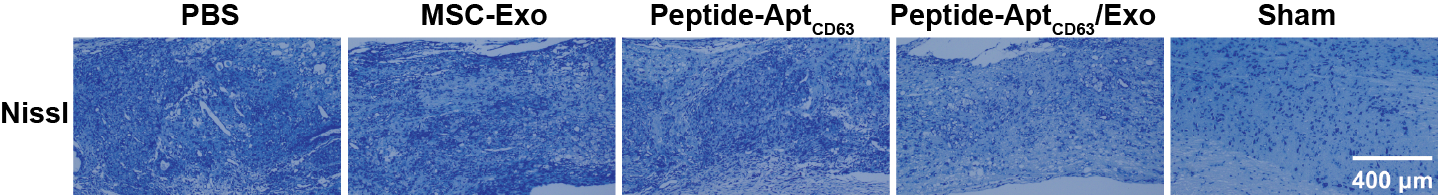


**Figure S30.** Nissl staining of the injury sites in different groups at 4 weeks post-surgery. Note that, Nissl bodies were inapparent in any of the SCI groups, suggesting that they had not yet fully matured at 4 weeks post-surgery.


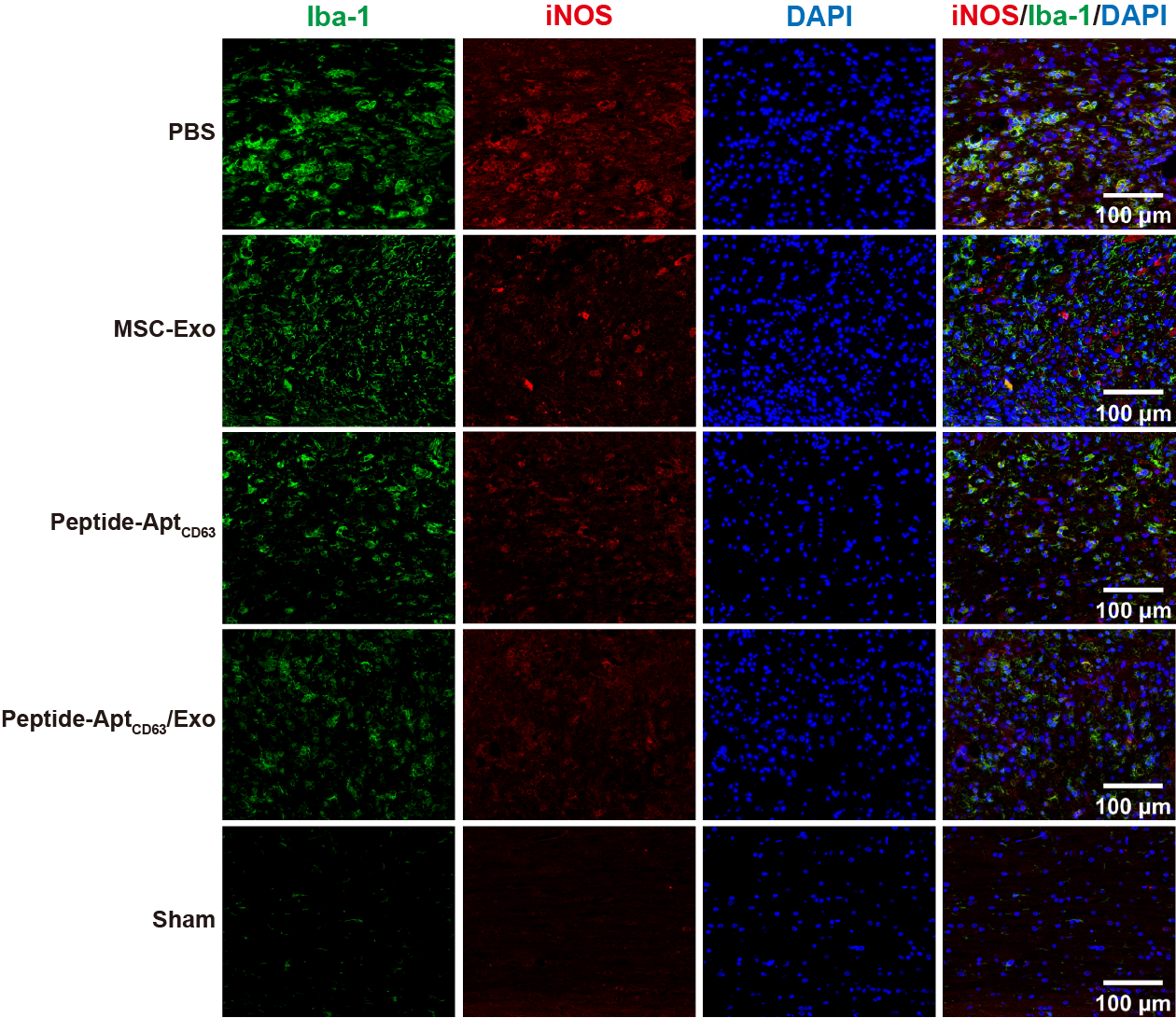


**Figure S31.** Immunofluorescence staining for iNOS and Iba-1 of the injury sites in different groups at 4 weeks post-surgery.


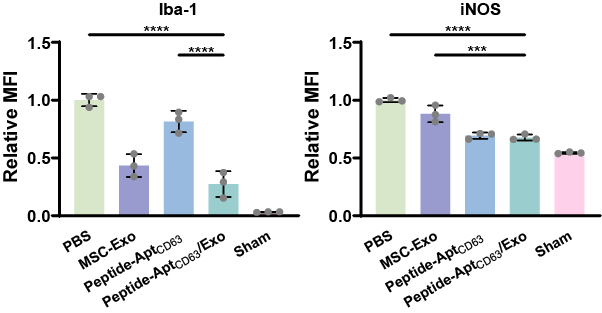


**Figure S32.** Relative MFI of immunostained Iba-1 and iNOS of the injury sites in Figure S31.


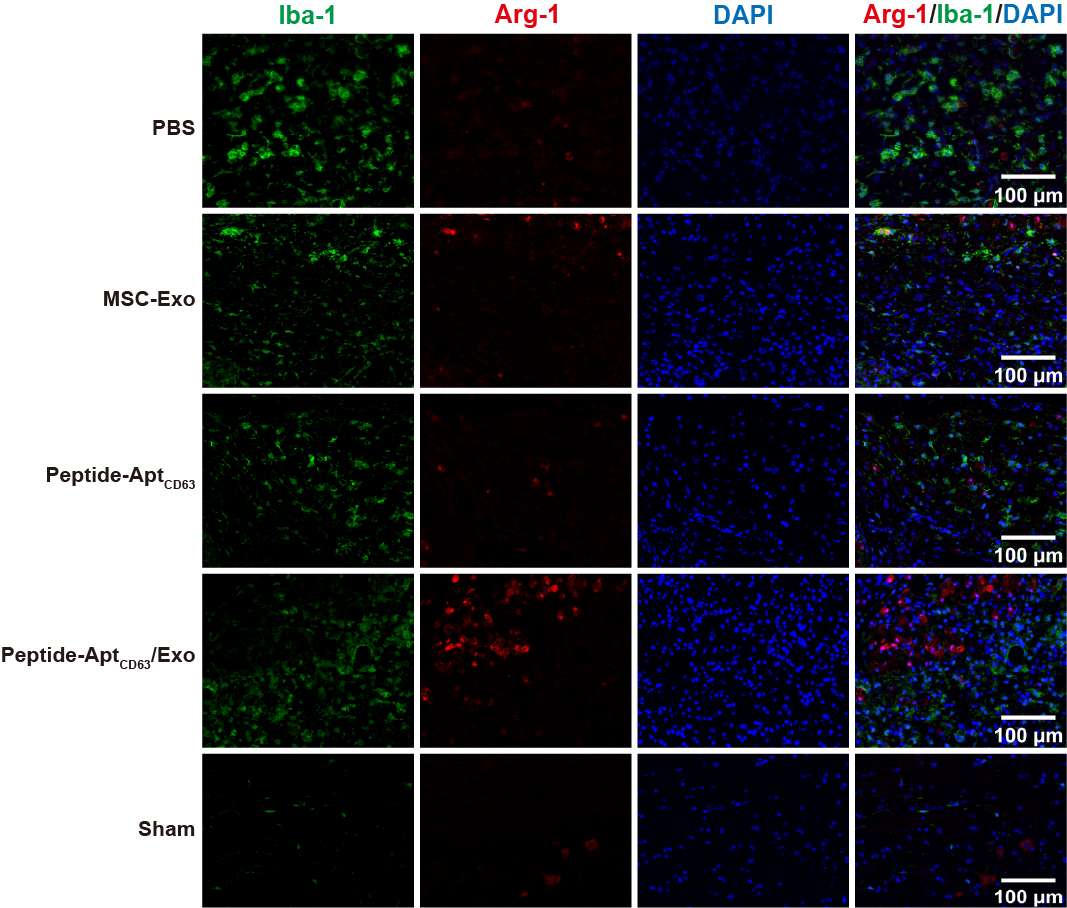


**Figure S33.** Immunofluorescence staining for Arg-1 and Iba-1 of the injury sites in different groups at 4 weeks post-surgery.


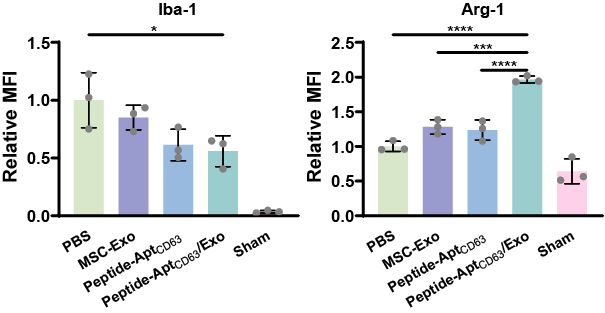


**Figure S34.** Relative MFI of immunostained Iba-1 and Arg-1 of the injury sites in Figure S33. Note that, immunofluorescence imaging (Figures S31, S33) and statistical analysis (Figures S32, S34) revealed attenuated Iba-1 signal intensity (indicative of microglial activation) and iNOS (an M1 polarization marker), as well as enhanced Arg-1 expression (an M2 polarization marker) in both MSC-Exo and Peptide-Apt_CD63_ groups versus PBS group. Notably, Peptide-Apt_CD63_/Exo microgels induced the most significant reduction in Iba-1 and iNOS expression concomitant with maximal Arg-1 induction, demonstrating their superior efficacy in reprogramming microglia from pro-inflammatory M1 to regenerative M2 phenotypes.


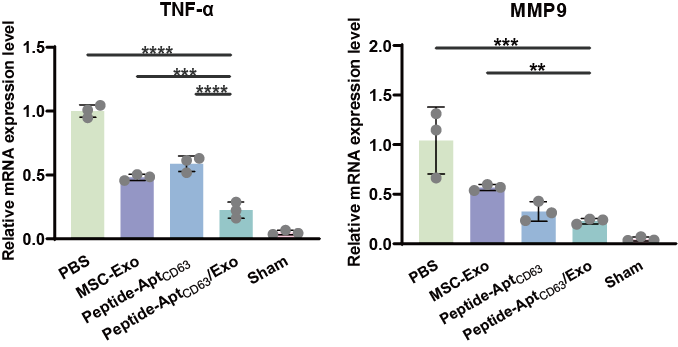


**Figure S35.** Relative mRNA expression levels of TNF-α and MMP9 of the injury sites in different groups at 4 weeks post-surgery.


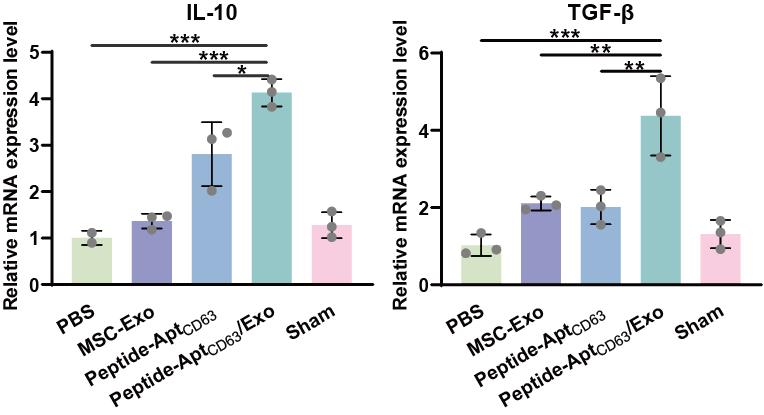


**Figure S36.** Relative mRNA expression levels of IL-10 and TGF-β of the injury sites in different groups at 4 weeks post-surgery. Note that, these results indicated that Peptide-Apt_CD63_/Exo microgels markedly downregulated the expression levels of pro-inflammatory TNF-α (~0.22-fold) and MMP9 (~0.23-fold) and simultaneously upregulated anti-inflammatory factors like IL-10 (~4.13-fold) and TGF-β (~4.37-fold) as compared to MSC-Exo (TNF-α, ~0.48-fold; MMP9, ~0.57-fold; IL-10, ~1.37-fold; TGF-β, ~2.12-fold) and Peptide-Apt_CD63_ (TNF-α, ~0.59-fold; MMP9, ~0.33-fold; IL-10, ~2.64-fold; TGF-β, ~2.01-fold) (Figures S35, S36). This enhanced immunomodulatory efficacy of Peptide-Apt_CD63_/Exo microgels indicates a superior capacity to reprogram the SCI microenvironment toward a repair-conducive state.


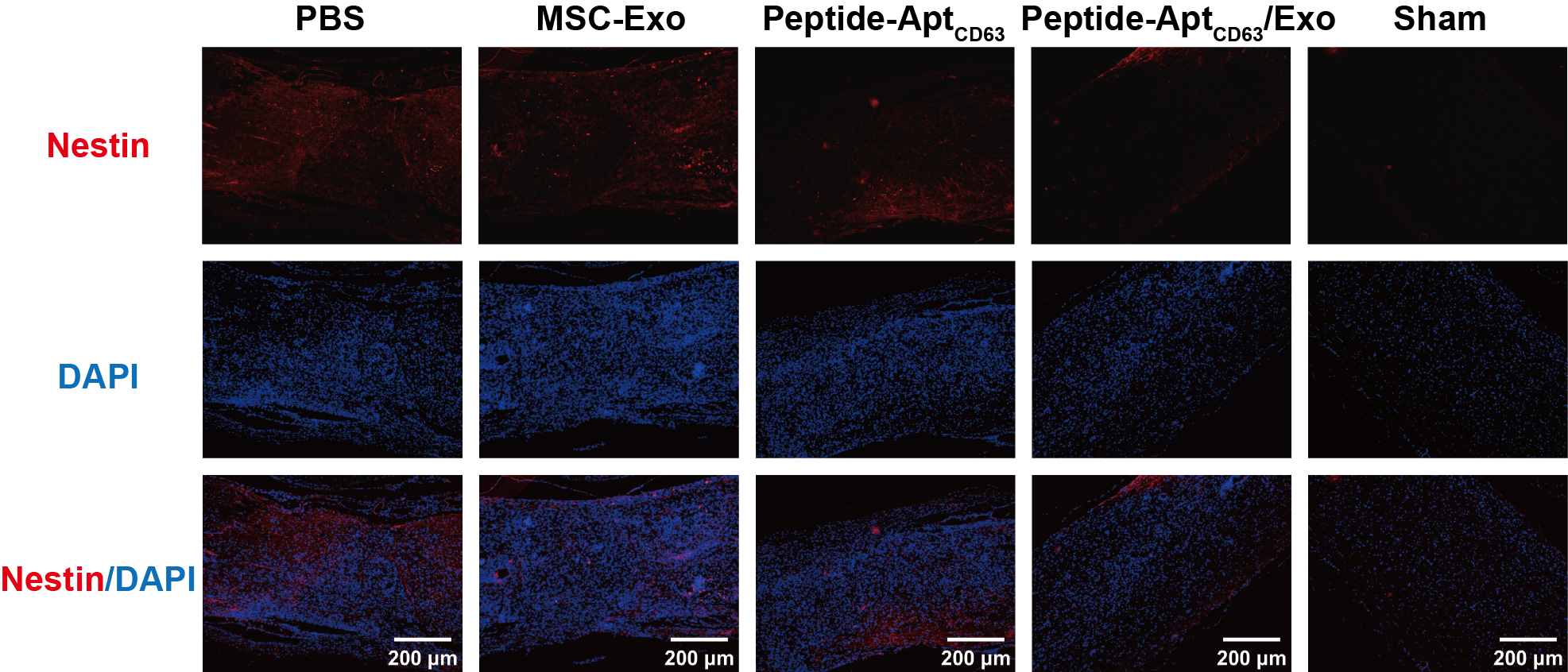


**Figure S37.** Immunofluorescence staining of Nestin of the injury sites in different groups at 4 weeks post-surgery.

**Figure S38.** Relative MFI of immunostained Nestin of the injury sites in Figure S37.

**Figure S39.** Relative mRNA expression level of Nestin of the injury sites in different groups at 4 weeks post-surgery. At 4 weeks post-surgery, both immunofluorescence staining (Figures S37, S38) and RT-qPCR results (Figure S39) revealed minimal differences in Nestin expression between the Exo and Peptide-Apt_CD63_ groups and the PBS group, indicating that few NSCs differentiated into neurons during the early repair stage. Conversely, the Peptide-Apt_CD63_/Exo group exhibited significantly lower Nestin expression, suggesting that microgels effectively promoted the differentiation of NSCs into neurons.


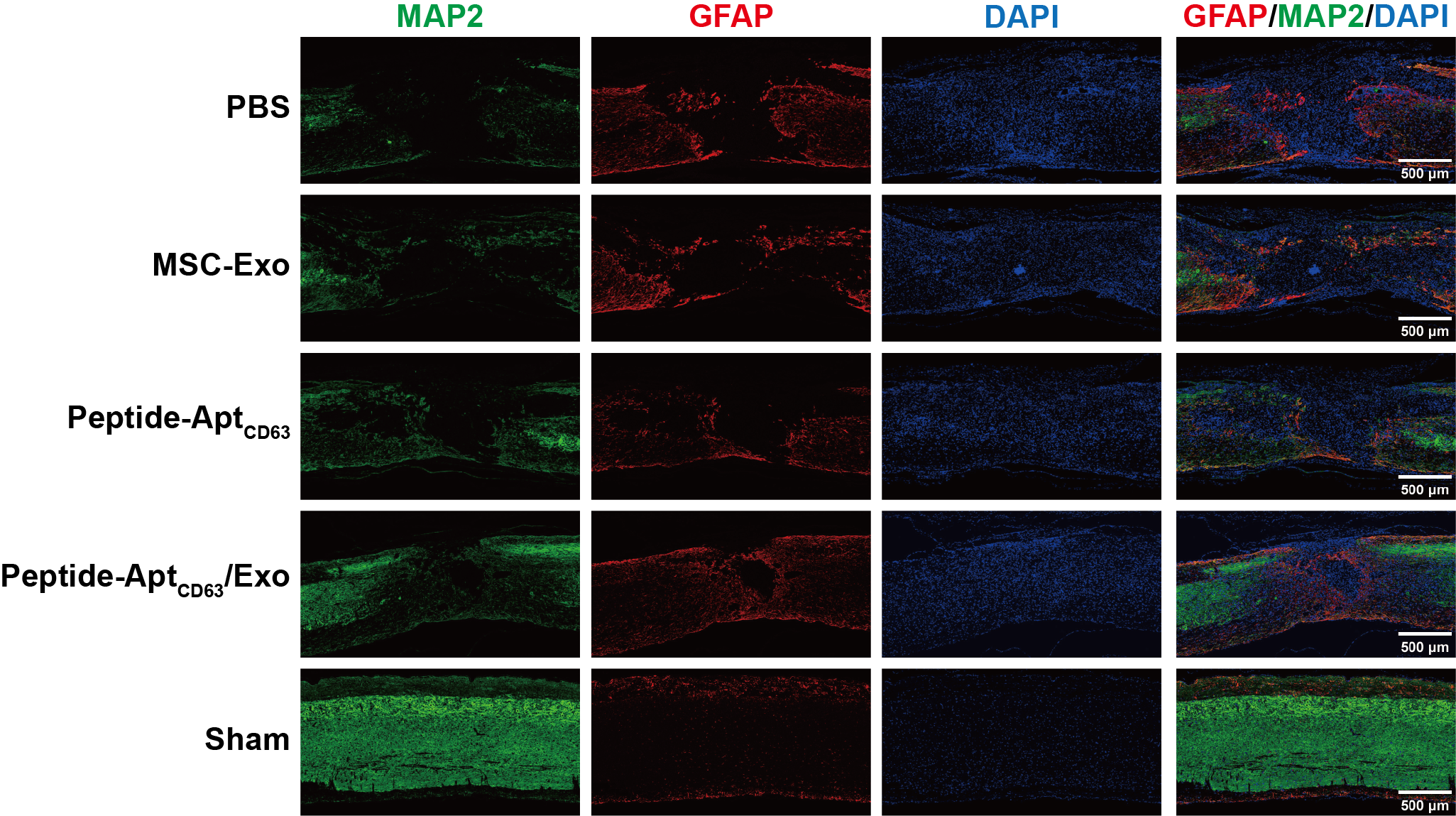


**Figure S40.** Immunofluorescence staining of MAP2 and GFAP of the injury sites in different groups at 4 weeks post-surgery.


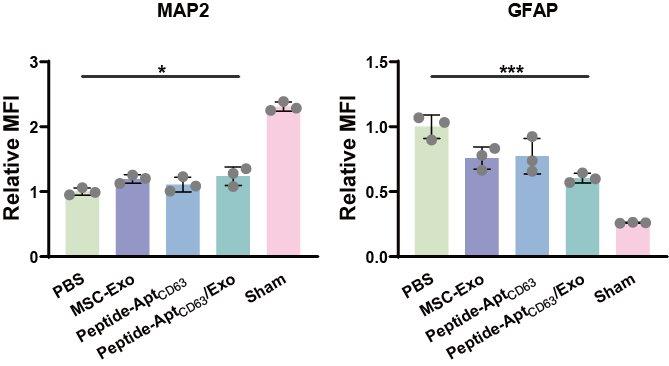


**Figure S41.** Relative MFI of immunostained MAP2 and GFAP of the injury sites in Figure S40.


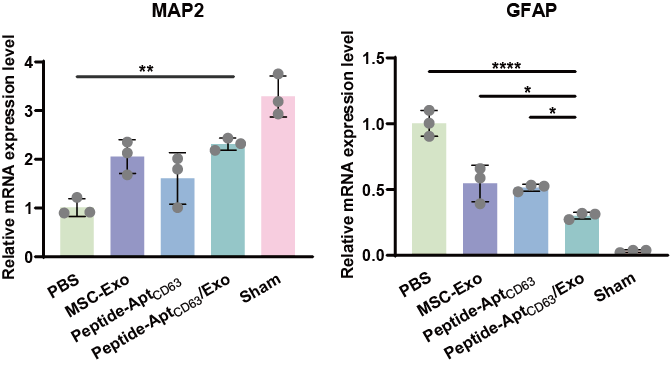


**Figure S42.** Relative mRNA expression levels of MAP2 and GFAP of the injury sites in different groups at 4 weeks post-surgery. At 4 weeks post-surgery, both immunofluorescence staining (Figures S40, S41) and RT-qPCR results (Figure S42) revealed that while all surgical groups displayed increased MAP2 expression and decreased GFAP expression compared to the PBS group, the Peptide-Apt_CD63_/Exo group exhibited higher MAP2 expression and lower GFAP expression relative to the Exo and Peptide-Apt_CD63_ groups. These findings suggest that microgels effectively promoted the differentiation of NSCs into neurons rather than astrocytes.


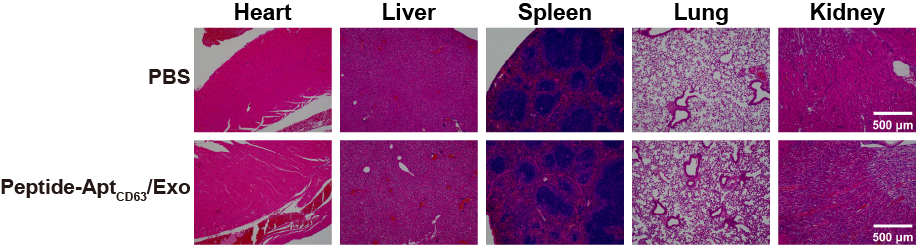


**Figure S43.** H&E staining of heart, liver, spleen, lung, and kidney collected from mice after treatment with Peptide-Apt_CD63_/Exo microgels.

**References**

69. R. Zhu, X. Zhu, Y. Zhu, Z. Wang, X. He, Z. Wu, et al., Immunomodulatory Layered Double Hydroxide Nanoparticles Enable Neurogenesis by Targeting Transforming Growth Factor-β Receptor 2, *ACS Nano* 15, no. 2 (2021): 2812-2830. http://doi.org/10.1021/acsnano.0c08727.
